# Platelet-derived extracellular vesicles inhibit ferroptosis and promote distant metastasis of nasopharyngeal carcinoma by upregulating ITGB3

**DOI:** 10.7150/ijbs.76162

**Published:** 2022-09-24

**Authors:** Fei Li, Ting Xu, Peiling Chen, Rui Sun, Chaoyi Li, Xin Zhao, Jinxin Ou, Jingyao Li, Taoshu Liu, Maozhen Zeng, Weizhong Zheng, Yunchen Lin, Le Yang, Zecang Li, Haisheng Chen, Qing Zhang

**Affiliations:** 1 State Key Laboratory of Biocontrol, School of Life Sciences, Sun Yat-sen University, Guangzhou, China.; 2 Department of Nasopharyngeal Carcinoma, Sun Yat-sen University Cancer Center; State Key Laboratory of Oncology in South China, Collaborative Innovation Center for Cancer Medicine, Guangzhou, China.; 3 Guangdong Key Laboratory of Nasopharyngeal Carcinoma Diagnosis and Therapy, Guangzhou, China.; 4 Institute of Sun Yat-sen University in Shenzhen, Shenzhen, China.

**Keywords:** nasopharyngeal carcinoma, platelets, extracellular vesicles, ferroptosis, ITGB3

## Abstract

Nasopharyngeal carcinoma (NPC) is a malignancy with high metastatic and invasive nature. Distant metastasis contributes substantially to treatment failure and mortality in NPC. Platelets are versatile blood cells and the number of platelets is positively associated with the distant metastasis of tumor cells. However, the role and underlying mechanism of platelets responsible for the metastasis of NPC cells remain unclear. Here we found that the distant metastasis of NPC patients was positively correlated with the expression levels of integrin β3 (ITGB3) in platelet-derived extracellular vesicles (EVs) from NPC patients (P-EVs). We further revealed that EVs transfer occurred from platelets to NPC cells, mediating cell-cell communication and inducing the metastasis of NPC cells by upregulating ITGB3 expression. Mechanistically, P-EVs-upregulated ITGB3 increased SLC7A11 expression by enhancing protein stability and activating the MAPK/ERK/ATF4/Nrf2 axis, which suppressed ferroptosis, thereby facilitating the metastasis of NPC cells. NPC xenografts in mouse models further confirmed that P-EVs inhibited the ferroptosis of circulating NPC cells and promoted the distant metastasis of NPC cells. Thus, these findings elucidate a novel role of platelet-derived EVs in NPC metastasis, which not only improves our understanding of platelet-mediated tumor distant metastasis, but also has important implications in diagnosis and treatment of NPC.

## Introduction

Nasopharyngeal carcinoma (NPC) is a malignant epithelial tumor with characteristic geographic and racial distribution, primarily in Southeast Asia and North Africa 1, 2. Radiotherapy or combined with chemotherapy is the mainstay of current therapies for patients with NPC 3. Although NPC is generally radiosensitive and the surgical treatment effect has greatly improved 4, NPC patients continue to show poor overall survival due to the rapid growth and high metastasizing tendency of NPC cells to regional lymph nodes and distant organs 2. Hematogenous metastasis is the main route of tumor metastasis to distal organs, which includes intravasation, transport in the blood, extravasation, and clonal expansion of single cancer cells in distant organs 5, 6. However, the molecular mechanism underlying the distant metastasis of NPC cells through blood circulation is not well understood. Exploring the molecular and biological changes that promote distant metastasis is of great value for generating new prognostic markers and developing novel treatment strategies for patients with NPC.

Platelets are anucleate blood cells generated from megakaryocytes that are required for hemostasis and thrombosis 7. Recently, emerging evidence has established that platelets are versatile cells involved in the pathological processes of tumor cell hematogenous metastasis, a process in which tumor cells transit from the primary tumor to distant organs via blood circulation 7, 8. Platelets enhance the hematogenous metastasis of tumor cells, a complex process that helps tumor cells escape immunosurveillance and arrest, adhere to the vessel wall, and extravasate 7. After tumor cells detach from the primary site and invade the blood circulation, platelets can adhere to tumor cells and protect them from shear force-induced tumor membrane damage and natural killer (NK) cytotoxicity 9-11. Platelets also mediate specific adhesive interactions between tumor cells and the vascular endothelium, thereby promoting circulating tumor cell arrest and adherence to vessels and facilitating tumor cell extravasation 7, 12, 13. However, the mechanism of direct or indirect information transmission between platelets and tumor cells is still not fully understood, and further investigation is required to reveal the specific markers of pathological information transfer in the distant metastasis of platelet-mediated tumor cells.

Extracellular vesicles (EVs) are sub-micrometer-sized membranous particles released from almost all cell types that have emerged as important mediators of intercellular communication by transferring bioactive cargo comprising proteins, lipids, and nucleic acids to recipient cells 14, 15. Studies have shown that this EVs-mediated cell-to-cell communication is involved in many pathological changes, including cancer progression and metastasis 15. In addition to promoting tumor progression by facilitating tumor proliferation and metastasis, tumor cell-derived EVs often lay the foundation for distant metastasis by preparing the premetastatic niche and determining organ targeting of tumor metastasis by carrying different proteins 16, 17. This includes the integrins α6β4 and α6β1 in EVs, which are associated with lung metastasis, whereas integrins αⅤβ5 and EGFR in EVs are linked to liver metastasis 16, 18. Platelets modulate the tumor microenvironment by stimulating tumor cell growth and angiogenesis 19. However, whether there is a similar dysregulation of platelet-derived EVs in tumor progression has not been adequately explored, and their role in platelet-mediated tumor cell distant metastasis is of great interest.

In this study, we demonstrated that platelet-derived EVs from NPC patients upregulated integrin β3 (ITGB3) expression and inhibited ferroptosis in NPC cells by upregulating SLC7A11 expression, thereby promoting the distant metastasis of NPC cells via blood circulation. These findings not only improve our understanding of platelets and EVs in NPC metastasis but also uncover potential novel therapeutic targets and biomarkers for NPC.

## Materials and methods

### Cell lines and patient samples

Human 6-10B and 5-8F cells were provided by the Sun Yat-sen University Cancer Center and cultured in RPMI-1640 medium supplemented with 10% fetal bovine serum (FBS, Gibco, USA). HEK293T cells were obtained from ATCC and were cultured in Dulbecco's Modified Eagle Medium supplemented with 10% FBS. The identity of all cell lines was confirmed using short tandem repeat profiling. LipoFilter (Hanbio, China) was used for transfection, according to the manufacturer's protocol. 6-10B-ITGB3 and 5-8F-ITGB3 cells were generated by transfecting 6-10B and 5-8F cells with ITGB3-expressing lentiviruses obtained from HEK293T cells co-transfected with pCDH-CMV-ITGB3-EF1-Puro, psPAX2, and pMD2.G. All clinical samples were obtained with informed consent from the Sun Yat-sen University Cancer Center and approved by the Hospital's Ethical Review Committees. Peripheral blood samples were collected from patients with NPC and healthy volunteers and used for subsequent platelet and platelet-derived EV isolation.

### Platelet-derived EVs isolation, identification, and internalization

A total of 10 ml peripheral blood was collected from NPC patients and healthy volunteers and used to prepare platelets as described previously 20. Fresh platelets were suspended in 5 ml of Tyrodes' buffer and incubated with 1 U/ml thrombin (Sigma, USA) for 1 h at 37℃. Subsequently, activated platelets were used for EVs collection as described previously 20, 21. Briefly, platelets and cell debris were removed by three rounds of 30-min centrifugation at increasing accelerations (200 × g, 1200 × g, and 20,000 × g) at room temperature. The remaining supernatant was filtered through a 0.22-μm membrane and EVs were pelleted twice at 120,000 × g for 2 h at 4℃ (SW 60Ti, Beckman, Germany). The isolated EVs were diluted in 100 µL phosphate-buffered saline (PBS) and quantified using a BCA protein assay kit (Thermo Scientific, USA). All isolated EVs were characterized according to the MISEV 2018 guidelines 22. The expression of ITGB3 in platelet-derived EVs were evaluated using enzyme-linked immunosorbent assay (ELISA) as described previously 23. Purified EVs derived from healthy volunteers (H-EVs) and NPC patients (P-EVs) were labeled with the PKH26 Red Fluorescent Cell Linker Kit (Sigma) according to the manufacturer's instructions, followed by co-culturing with 6-10B and 5-8F cells for 2 h. The cells were washed twice with PBS and fixed in 4% paraformaldehyde. Cell nuclei were stained with Hoechst 33342 and observed using an LSM880 laser confocal microscope (Zeiss, Germany). The stereoscopic structure of the cells was visualized using differential interference contrast.

### *In vitro* studies

Generation of ITGB3 and SLC7A11 knockout cells, western blotting, co-immunoprecipitation (Co-IP), nanoparticle tracking analysis (NTA), immunofluorescence, cell migration, invasion assay, clone formation, adhesion, transendothelial migration assays, 3D spheroid cultures, total RNA isolation and expression analysis (Gene Expression Omnibus accession number GSE196879), flow cytometry analysis, intracellular reactive oxygen species (ROS) detection, GSH/GSSG detection, cell viability assay, lipid peroxidation assay, transmission electron microscopy, mitochondrial membrane potential assay, intracellular iron (Fe^2+^) measurement, and chromatin immunoprecipitation and quantitative polymerase chain reaction (ChIP-qPCR) were performed using standard procedures (see Supplementary Materials for details).

### Animal models

Five-week-old BALB/c nude mice were maintained at the Laboratory Animal Center of Sun Yat-sen University. The animal studies were authorized by the Institutional Animal Care and Use Committee of the Sun Yat-sen University (Approval number: SYSU-IACUC-2020-B0164). All animal experiments were strictly implemented in compliance with the ARRIVE guidelines. Mice were randomly divided into four groups, and NPC ascitic tumors were generated by co-injecting 1×10^6^ 6-10B or 5-8F cells with PBS or P-EVs (10 µg) into the abdominal cavity of BALB/c nude mice. After 20 days, the mice were sacrificed and the ascitic tumor cells were collected for western blotting and flow cytometry. For the NPC hematogenous metastasis model, 1×10^6^ 6-10B or 5-8F cells were mixed with or without P-EVs (10 µg) in 150 µL PBS and injected into the tail vein of BALB/c nude mice. Two weeks after inoculation, the transplanted mice were used for circulating tumor cell isolation and histological or Kaplan-Meier survival analysis, as described previously 21, 24.

### Statistical analysis

All data are reported as the mean ± SD of at least three independent experiments. Statistical analysis was performed using the paired or unpaired two-tailed Student's t-test for two groups or one-way analysis of variance for comparisons between more than two groups. Kaplan-Meier survival curves for mice and P-values were obtained using the log-rank (Mantel-Cox) test. *^⁎^P* < 0.05, *^⁎⁎^P* < 0.01, and *^⁎⁎⁎^P* < 0.001 were considered statistically significant.

## Results

### Platelet-derived EVs of NPC patients mediate platelet-tumor cell communication and promote metastasis of NPC cells

**Figure 1:**
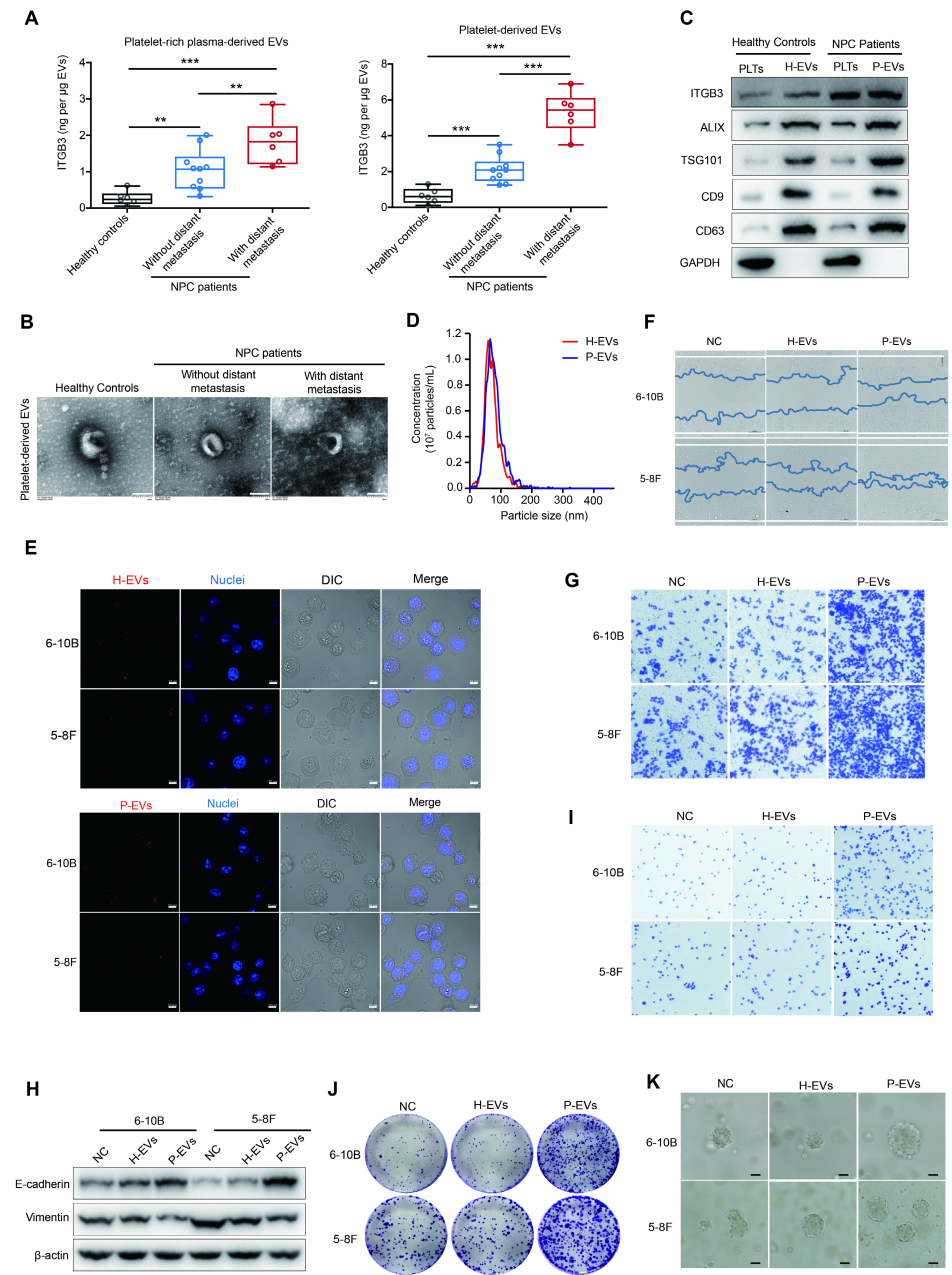
** Platelet-derived EVs of NPC patients promote metastasis of NPC cells.** (**A**) ELISA of ITGB3 on EVs from platelet-rich plasma and platelets of healthy volunteers (n = 6), NPC patients without distant metastasis (n = 10), and NPC patients with distant metastasis (n = 6). (**B**) Transmission electron microscopy analysis of EVs from platelets of healthy volunteers, NPC patients without distant metastasis, and NPC patients with distant metastasis (Scale bar, 100 nm). (**C**) Western blot of ITGB3 and EVs markers (ALIX, TSG101, CD9, and CD63) in platelets and platelet-derived EVs of healthy volunteers and NPC patients. PLTs: Platelets. (**D**) Nanoparticle tracking analysis to determine size distribution and total number of EVs isolated from the same platelet count of healthy volunteers and NPC patients. (**E**) Confocal microscopy image showing the internalization of PKH26-labeled H-EVs and P-EVs (red) by 6-10B and 5-8F cells. Hoechst 33342 was used to stain nuclei (blue). Differential interference contrast (DIC) was used to observe the stereoscopic structure of cells (Scale bar, 10 µm). (**F**) Wound healing assay showing effects of H-EVs and P-EVs treatment on 6-10B and 5-8F cells migration. (**G**) Matrigel invasion assay was performed to measure the effects of H-EVs and P-EVs on 6-10B and 5-8F cells invasion. (**H**) Western blot analysis for EMT markers E-cadherin and Vimentin in 6-10B and 5-8F cells after treatment with H-EVs and P-EVs. (**I**) Adhesion assay showing effects of H-EVs and P-EVs internalization on 6-10B and 5-8F cells adhesion. (**J**) Clonogenic assays were performed to reveal the effects of H-EVs and P-EVs on clone formation capacity of 6-10B and 5-8F cells. (**K**) 3D spheroid forming assay determined the effect of H-EVs and P-EVs on 6-10B and 5-8F tumorsphere propagation (Scale bar, 50 µm). Experiments were performed in triplicate.

Platelets are the major blood component involved in hemostasis and promote the distant metastasis of tumor cells via blood circulation 7, 8. However, the mechanism underlying platelet-tumor cell interaction remains unclear and is still an area of active investigation. By analyzing the BBCancer 25, a blood-based biomarkers of cancers database, we found that the expression level of integrin β3 (ITGB3) was significantly increased in the circulating tumor cells, blood, and EVs from tumor patients than in those from normal controls (**Figure S1A**). Further analysis by ELISA revealed that ITGB3 expression level was significantly higher in platelet-rich plasma- and platelet-derived EVs from NPC patients than in those from healthy volunteers, and the expression level of ITGB3 in platelet-derived EVs is positively correlated with the distant metastasis of NPC patients (**Figure 1A**). However, the transmission electron microscopy observations revealed that there were no differences of the structure, size, and morphology of platelet-derived EVs among healthy volunteers and NPC patients without distant metastasis and NPC patients with distant metastasis (**Figure 1B**). Considering that EVs are tiny vesicles secreted by various cells that can mediate intercellular communication by delivering proteins, lipids, and nucleic acids, we collected and isolated platelet-derived EVs from NPC patients (P-EVs) and healthy volunteers (H-EVs) to explore whether platelet-derived EVs mediate platelet-tumor communication. Western blotting also confirmed that ITGB3 exhibited significantly increased expression in P-EVs compared to H-EVs (**Figure 1C**). After characterized by detecting enrichment of non-membrane (ALIX and TSG101) and membrane (CD9 and CD63) EV markers, and determined the size distribution of EV isolates using NTA (**Figure 1C and Figure 1D**), H-EVs and P-EVs were labeled with PKH26 and co-cultured with 6-10B or 5-8F cells. Confocal microscopy showed that both PKH26-labeled H-EVs and P-EVs were internalized by 6-10B and 5-8F cells during a 2-h incubation (**Figure 1E**).

However, only the internalization of P-EVs, but not H-EVs, promoted the migration and invasion of NPC cells (**Figure 1F and Figure 1G**), indicating that the uptake of P-EVs mediate platelet-NPC cell communication and promote metastasis of tumor cells in NPC patients.

Notably, P-EVs treatment reduced vimentin levels and increased E-cadherin levels in 6-10B and 5-8F cells (**Figure 1H**), which is P-EVs treatment reversed the epithelial-mesenchymal transition (EMT), a pathological transformation that mediates the conspicuous downregulation of epithelial marker expression and loss of intercellular junctions. Subsequently, double immunofluorescence staining further confirmed that treatment with P-EVs led to an increase in E-cadherin levels and a decrease in vimentin levels in 6-10B and 5-8F cells (**Figure S1B**), suggesting that P-EVs may induce mesenchymal-epithelial transition (MET) and lead to metastatic colonization by enhancing intercellular adhesion at metastatic sites. Correspondingly, following treatment with P-EVs, focal adhesion and cellular clonogenic ability were enhanced compared to that in the H-EVs-treated and untreated groups in NPC cells (**Figure 1I and Figure 1J**). Z-stack confocal imaging showed that P-EVs-treated 6-10B-GFP and 5-8F-GFP cells readily migrated across the HMEC-1 endothelial cell layers compared to untreated NPC cells (**Figure S1C**). In line with the results obtained from 2D cell cultures, 3D spheroid cultures also demonstrated that P-EVs, but not H-EVs, treatment increased spheroid dimensions in NPC cells and induced the MET process in NPC spheroids recovered from 3D spheroid culture (**Figure 1K and Figure S1D**). Overall, these results suggest that EVs secreted by platelets mediate the communication of information between platelets and NPC cells, and therefore promote distant metastasis and induce the formation of secondary metastatic foci of NPC cells.

### P-EVs mediate ITGB3 expression upregulation to promote metastasis of NPC cells

**Figure 2:**
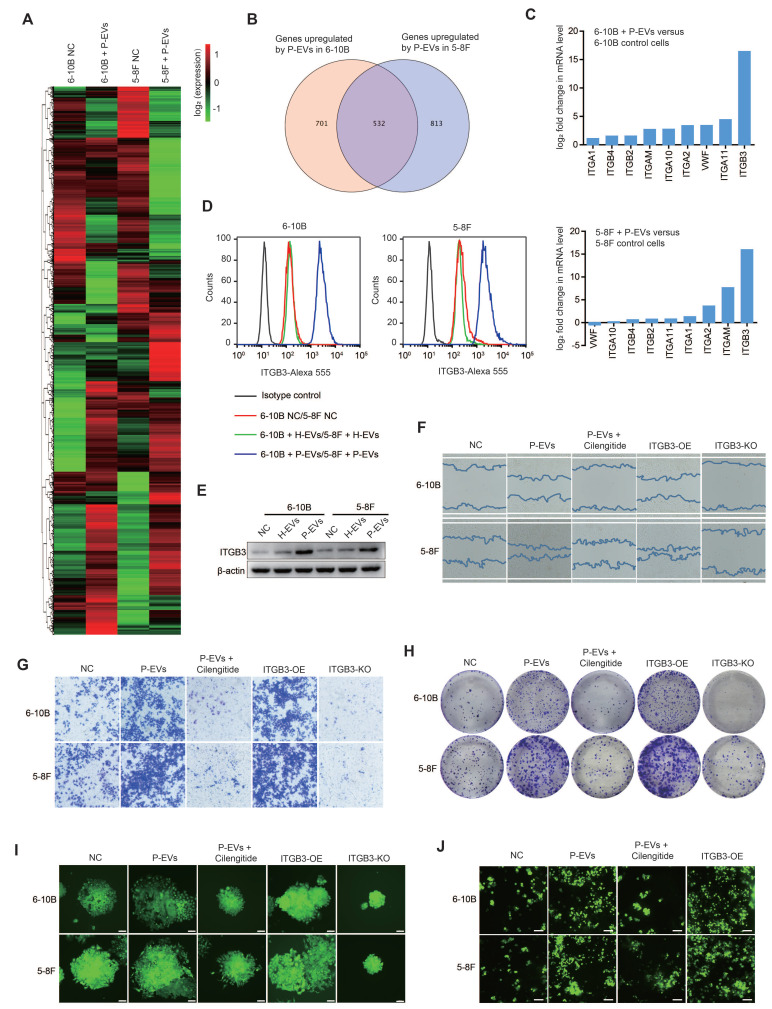
** P-EVs promote NPC metastasis by upregulating ITGB3.** (**A**) Heatmap displaying the hierarchical clustering of genes in 6-10B and 5-8F cells in response to P-EVs treatment. Gene expressions are indicated by the color intensities of red (upregulated) or green (downregulated). (**B**) Expression of potential genes upregulated by P-EVs treatment in 6-10B and 5-8F cells; numbers indicate quantity of genes in each differentially expressed genes (DEGs) subset. (**C**) Column diagram represents expression of integrin genes following P-EVs treatment in 6-10B and 5-8F cells. (**D**) Flow cytometry of cell surface expression of ITGB3 on 6-10B and 5-8F cells treatment with H-EVs or P-EVs. Mouse IgG was used as an isotype control. (**E**) Western blot of ITGB3 expression in 6-10B and 5-8F cells treatment with H-EVs or P-EVs. (**F-G**) Wound healing assay (**F**) and matrigel invasion assay (**G**) showing cell migration and invasion of P-EVs-treated, P-EVs and cilengitide co-treated, ITGB3 overexpressed, and ITGB3 knockout 6-10B and 5-8F cells (Scale bar, 200 µm). KO: Knockout; OE: Overexpression. (**H-I**) Clone formation capacity (**H**) and single-cell-derived clone growth (Scale bar, 100 µm) (**I**) of P-EVs-treated, P-EVs and cilengitide-co-treated, ITGB3-overexpressed, and ITGB3 knockout 6-10B and 5-8F cells using clonogenic assays. (**J**) Cell adhesion of P-EVs-treated, P-EVs and cilengitide-co-treated, and ITGB3-overexpressed 6-10B and 5-8F cells using a parallel plate flow adhesion assay (Shear stress, 5 dynes/cm^2^; Scale bar, 36.71 µm).

To shed light on the mechanism underlying P-EVs-induced NPC metastasis, we performed high-throughput RNA-Seq analyses to reveal differentially expressed genes (DEGs) in NPC cells treated with or without P-EVs. Interestingly, we observed a significant overlap in the expression of integrins that were altered in 6-10B and 5-8F upon P-EVs treatment (**Figure 2A, 2B and Figure S2A**). Of these, consistent with the clinical data that ITGB3 was highly expressed in P-EVs, ITGB3 expression exhibited the most distinct upregulation in P-EVs-treated NPC cells (**Figure 2C and Figure S2B**), which was further confirmed by flow cytometry and western blot analysis that treatment with P-EVs instead of H-EVs upregulated the expression of ITGB3 in NPC cells (**Figure 2D and Figure 2E**), indicating that P-EVs induced the upregulation of ITGB3 at both the transcript and protein level.

Considering that integrins are the major receptor family in platelets and have been associated with cancer progression and metastasis 26, 27, we further explored the interrelationship between P-EVs-induced ITGB3 upregulation and NPC progression and metastasis by constructing ITGB3-overexpressing and knockout 6-10B and 5-8F cells (**Figure S2C**). We found that both P-EVs treatment and ITGB3 overexpression enhanced the migration of NPC cells, but ITGB3 knockout and treatment with cilengitide, an ITGB3 inhibitor, impaired the P-EVs-induced migration of NPC cells (**Figure 2F**). Parallelly, P-EVs treatment and ITGB3 overexpression promoted the invasion of NPC cells, which was reversed by cilengitide treatment or ITGB3 knockout (**Figure 2G**). Moreover, cilengitide treatment and ITGB3 knockout inverted the P-EVs- and ITGB3-enhanced single-cell-derived clone growth and clonogenic ability of NPC cells (**Figure 2H and Figure 2I**). More importantly, using a parallel plate flow adhesion assay, we demonstrated that P-EVs increased the adhesion of NPC cells under conditions of 5 dynes/cm^2^ shear forces, but this adhesion was abolished by cilengitide (**Figure 2J**), suggesting that P-EVs may promote the adhesion of blood-circulating NPC cells to mediate the formation of secondary metastatic foci by inducing ITGB3 expression. Collectively, these results indicate that P-EVs induce the upregulation of ITGB3 expression in NPC cells and therefore, have a novel metastasis-promoting effect.

### P-EVs-upregulated ITGB3 expression inhibits ferroptosis in NPC cells

**Figure 3:**
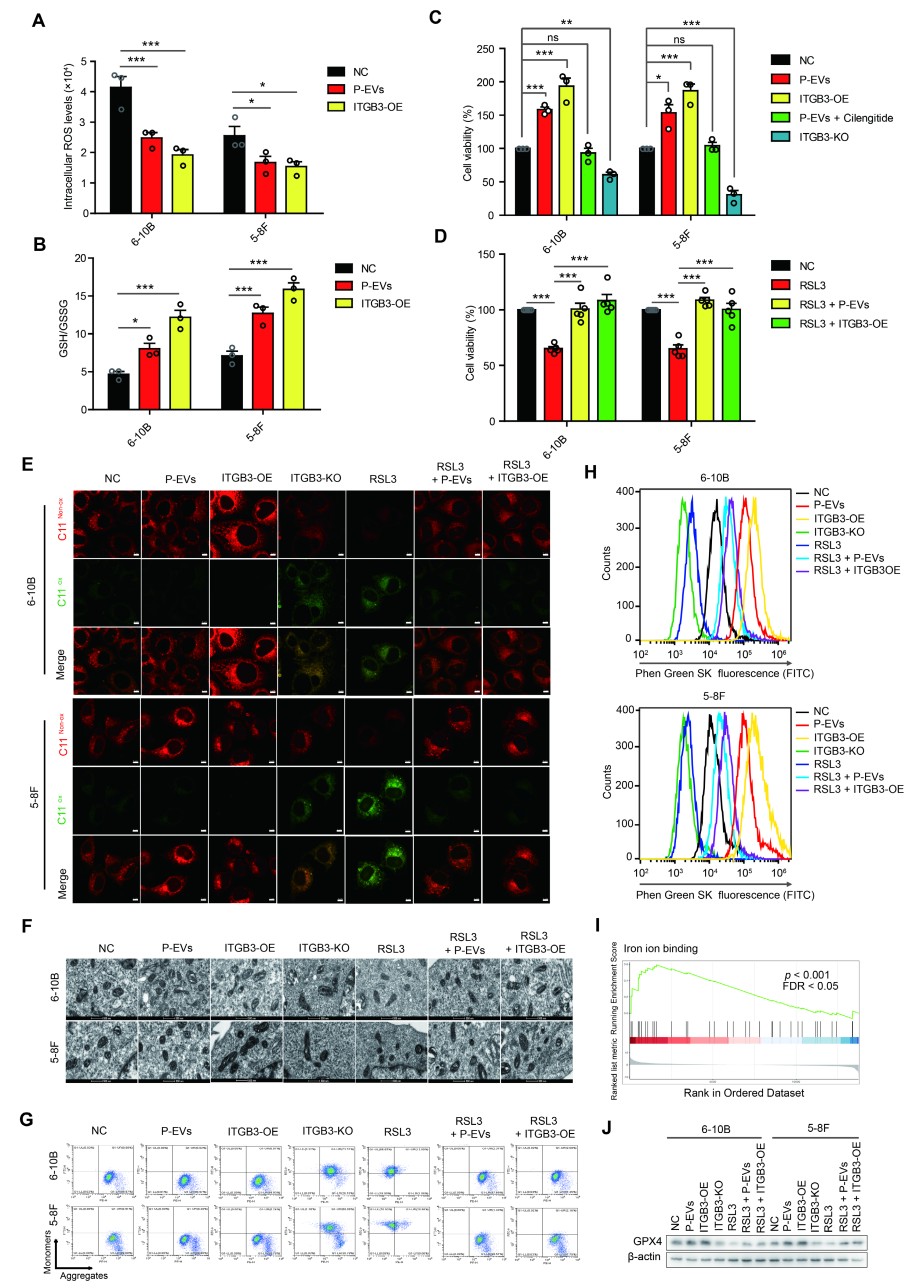
** P-EVs upregulate ITGB3 expression to inhibit ferroptosis in NPC cells.** (**A-B**) Intracellular ROS levels (**A**) and cellular GSH/GSSG ratio (**B**) in P-EVs-treated and ITGB3-overexpressed 6-10B and 5-8F cells by fluorescence microplate reader. (**C**) Cell viability of P-EVs-treated, P-EVs and cilengitide-co-treated, ITGB3-overexpressed, and ITGB3 knockout 6-10B and 5-8F cells using CCK-8 assay. (**D**) Cell viability of RSL3-treated, RSL3 and P-EVs-co-treated, ITGB3 overexpression combined with RSL3 treated 6-10B and 5-8F cells. (**E**) Confocal imaging showed the lipid peroxidation activity (Green) using C11 BODIPY 581/591 in P-EVs-treated, ITGB3-overexpressed, ITGB3 knockout, RSL3-treated, RSL3 and P-EVs-co-treated, and ITGB3 overexpression combined with RSL3 treated 6-10B and 5-8F cells. C11^Non-ox^ = non-oxidized C11 (Red), C11^Ox^ = oxidized C11 (Green) (Scale bar, 5 µm). (**F**) Mitochondrial morphology of P-EVs-treated, ITGB3-overexpressed, ITGB3 knockout, RSL3-treated, RSL3 and P-EVs-co-treated, and ITGB3 overexpression combined with RSL3 treated 6-10B and 5-8F cells were observed by transmission electron microscopy (Scale bar, 500 nm). (**G**) Flow cytometry analysis of mitochondrial membrane potential in P-EVs-treated, ITGB3-overexpressed, ITGB3 knockout, RSL3 (10 µM)-treated, RSL3 and P-EVs-co-treated, and ITGB3 overexpression combined with RSL3 treated 6-10B and 5-8F cells using JC-10. (**H**) Flow cytometry analysis of intracellular free iron (Fe^2+^) levels in P-EVs-treated, ITGB3-overexpressed, ITGB3 knockout, RSL3-treated, RSL3 and P-EVs-co-treated, and ITGB3 overexpression combined with RSL3 treated 6-10B and 5-8F cells using the fluorescent indicator Phen Green SK. (**I**) Select enrichment plots highlighting the enriched iron ion binding-related gene identified using GSEA in P-EVs-treated NPC cells. (**J**) Western blot analysis of GPX4 expression in P-EVs-treated, ITGB3-overexpressed, ITGB3 knockout, RSL3 (10µM)-treated, RSL3 and P-EVs-co-treated, and ITGB3 overexpression combined with RSL3 treated 6-10B and 5-8F cells. Experiments were repeated at least thrice. Data represent the mean ± SD (^*^p<.05; ^**^p<.01; ^***^p<.001).

Circulating cancer cells in the blood experience high levels of oxidative stress and therefore inhibit the distant metastasis of these cancer cells 28. As ferroptosis is an iron-dependent cell death process driven by the accumulation of lipid ROS, and is also a critical regulator of tumor growth 29, 30, we first tested the intratumoral ROS levels and found that both P-EVs treatment and ITGB3 overexpression reduced the ROS levels in NPC cells (**Figure 3A**). Conversely, the GSH/GSSG ratio, a cellular antioxidant system that prevents oxidative damage, was elevated by P-EVs treatment and ITGB3 overexpression (**Figure 3B**), suggesting that P-EVs-upregulated ITGB3 expression likely inhibits oxidative stress and therefore antagonizes ferroptosis in NPC cells. To test this possibility, cell viability of NPC cells was detected by the CCK-8 assay, and the results indicated that P-EVs treatment and ITGB3 overexpression increased the viability of NPC cells, but this enhancement was abolished by cilengitide and ITGB3 knockout (**Figure 3C**). Treatment with buthionine sulfoximine (BSO), a GSH synthesis inhibitor, and elesclomol, a ROS inducer, reversed the P-EVs- and ITGB3-enhanced NPC cell viability (**Figure S3A**). However, P-EVs treatment and ITGB3 overexpression suppressed RSL3, a ferroptosis inducer, -inhibited cell viability of NPC (**Figure 3D**). Using BODIPY™ 581/591 C11 as a lipid peroxidation probe, we demonstrated that although ITGB3 knockout and RSL3 treatment increased lipid peroxidation, which is a hallmark of ferroptosis, P-EVs treatment and ITGB3 overexpression inhibited RSL3-induced lipid peroxidation in NPC cells (**Figure 3E and Figure S3B**). Transmission electron microscopy and mitochondrial membrane potential analysis also showed that ITGB3 knockout and RSL3 treatment caused mitochondrial damage and led to the depolarization of the mitochondria and the decrease of the mitochondrial membrane potential, but P-EVs treatment and ITGB3 overexpression reversed this damage and depolarization (**Figure 3F and Figure 3G**). The intracellular free iron (Fe^2+^) fluorescent indicator Phen Green SK, whose fluorescence was quenched by iron, showed that ITGB3 knockout and RSL3 treatment triggered an increase in intracellular free iron (Fe^2+^) levels in NPC cells, which was reversed upon P-EVs treatment and ITGB3 overexpression (**Figure 3H and Figure S3C**). Conversely, gene set enrichment analysis (GSEA) of DEGs revealed that the regulation of iron ion binding-related pathways was significantly enriched in P-EVs-treated NPC cells (**Figure 3I**). Moreover, the expression of GPX4, the negative regulatory protein for ferroptosis, was significantly increased after P-EVs treatment and ITGB3 overexpression. P-EVs treatment and ITGB3 overexpression reversed the RSL3 decreased GPX4 expression (**Figure 3J**). Consistently, comparative transcriptome analyses indicated that P-EVs treatment upregulated the majority of ferroptosis-suppressing genes, including GPX4 and ATF4, and downregulated the ferroptosis-promoting genes ACSL4 and POR in 6-10B and 5-8F cells (**Figure S3D**). Taken together, these findings strongly suggest that P-EVs-mediated upregulation of ITGB3 antagonizes ferroptotic cell death in NPC cells.

### P-EVs-upregulated ITGB3 promotes SLC7A11 expression by enhancing protein stability and activating MAPK/ERK pathway

**Figure 4:**
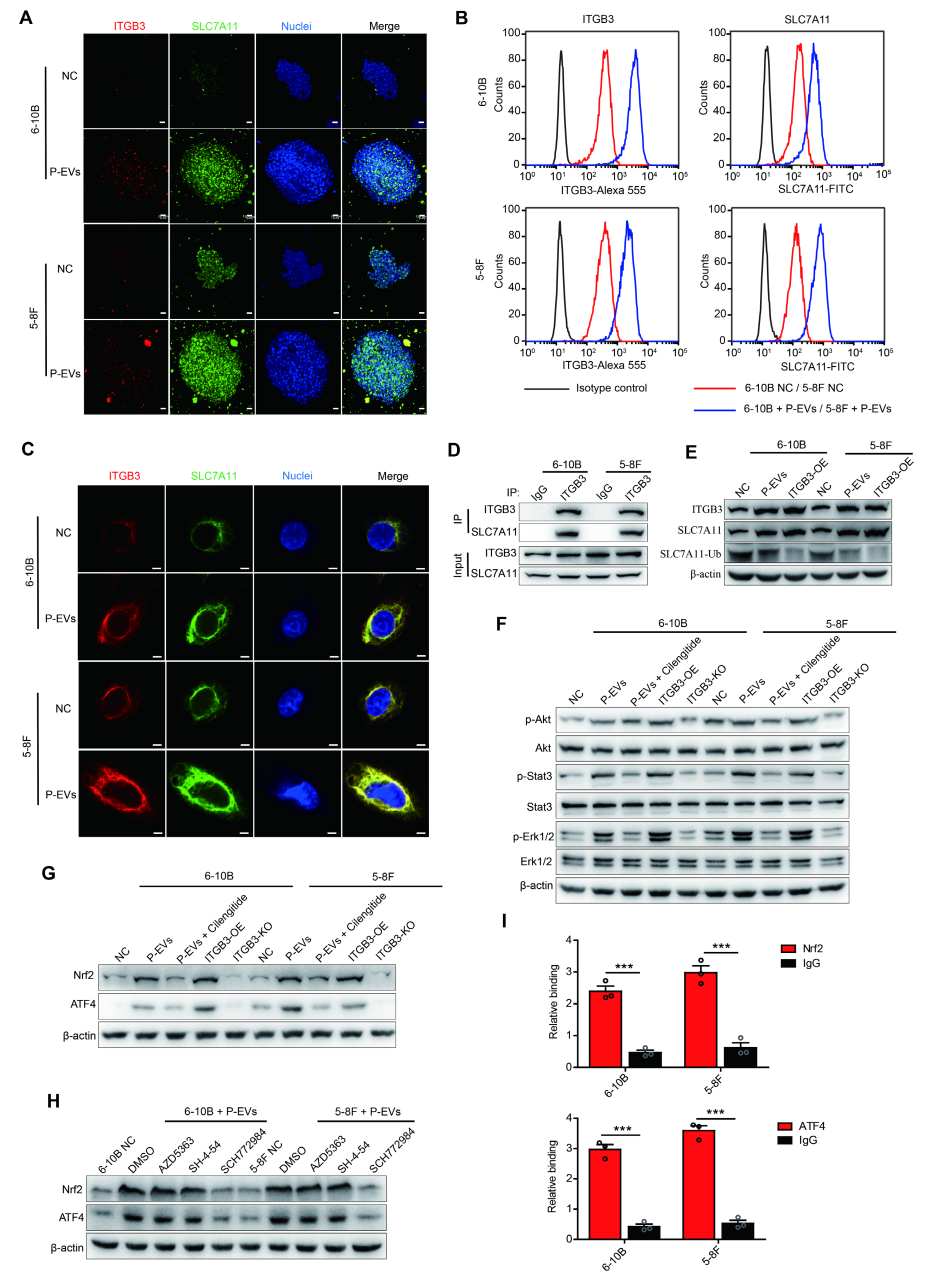
** P-EVs-upregulated ITGB3 promotes SLC7A11 expression by enhancing protein stability and activating MAPK/ERK pathway.** (**A**) Representative immunofluorescence of ITGB3 and SLC7A11 in 6-10B and 5-8F spheroids released from 3D spheroid culture. Images are maximum intensity z-projections from a confocal z-stack. Hoechst 33342 was used to stain the nuclei of cells (blue) (Scale bar, 20 µm). (**B**) Flow cytometric analysis of cell surface expression of ITGB3 and SLC7A11 on 6-10B and 5-8F cells treatment with or without P-EVs. Mouse IgG and Rabbit IgG were used as isotype control, respectively. (**C**) Immunofluorescence microscopy of ITGB3 and SLC7A11 in 6-10B and 5-8F pretreated with P-EVs. Hoechst 33342 was used to stain the nuclei of cells (blue) (Scale bar, 5 µm). (**D**) 6-10B and 5-8F cells were lysed with RIPA buffer. The interaction between ITGB3 and SLC7A11 was detected by IP using ITGB3 antibody, followed by detection using ITGB3 and SLC7A11 western blotting antibodies. Rabbit IgG was used as isotype control. (**E**) Western blot analysis of ITGB3, SLC7A11, and ubiquitinylated SLC7A11 in P-EVs-treated and ITGB3-overexpressed 6-10B and 5-8F cells. Abundance of ubiquitinylated SLC7A11 was determined via co-immunoprecipitation with an anti- SLC7A11 antibody as the immunoprecipitant. (**F-G**) Western blot analysis of phospho-Akt, Akt, phospho-Stat3, Stat3, phospho-Erk1/2, Erk1/2 (**F**), Nrf2, and ATF4 (**G**) in P-EVs-treated, P-EVs and cilengitide-co-treated, ITGB3-overexpressed, and ITGB3 knockout 6-10B and 5-8F cells. (**H**) Western blot analysis of Nrf2 and ATF4 in P-EVs-treated 6-10B and 5-8F cells pretreated with DMSO control, AZD5363, SH-4-54, and SCH772984. (**I**) ChIP-qPCR analysis of Nrf2 and ATF4 binding to the SLC7A11 promoter region in 6-10B and 5-8F cells. ChIP assay was performed using anti-Nrf2 or anti-ATF4 antibody. Rabbit IgG was used as isotype control. ChIP-qPCR enrichment was shown as the percentage of input DNA. Experiments were repeated at least thrice. Data represent the mean ± SD (^***^p<.001).

To investigate the potential mechanisms underlying P-EVs-upregulated ITGB3 expression promoting NPC cell metastasis and antagonizing ferroptosis, we detected the expression of SLC7A11, a subunit unique to the xc(-) system, which modulates ferroptosis and tumor metastasis by maintaining the intracellular redox balance of cancer cells 31. Using 3D spheroid cultures and immunofluorescence, we observed that except for increased spheroid dimensions, P-EVs treatment also upregulated SLC7A11 expression concurrent with the increase in ITGB3 expression (**Figure 4A**). Consistently, flow cytometry analysis also demonstrated that P-EVs treatment upregulated SLC7A11 expression in NPC cells parallelly with an increase in ITGB3 expression (**Figure 4B**). Meanwhile, we discovered that there was a significant degree of co-localization between ITGB3 and SLC7A11, and the expression increased synchronously (**Figure 4C**). Co-IP confirmed the strong protein-protein interaction between ITGB3 and SLC7A11 proteins (**Figure 4D**). However, although P-EVs upregulated ITGB3 and synchronously promoted the expression of SLC7A11, the ubiquitination level of SLC7A11 decreased significantly (**Figure 4E**), indicating that ITGB3 interacts with SLC7A11 and thereby enhances the protein stability of SLC7A11.

In addition, treatment with P-EVs and ITGB3 overexpression induced abnormal activation of Akt, Stat3, and Erk1/2 signaling in 6-10B and 5-8F cells, but this activation was abolished by cilengitide treatment or ITGB3 knockout (**Figure 4F**). Parallelly, both P-EVs treatment and ITGB3 overexpression led to elevated expression of transcription factors Nrf2 and ATF4 in 6-10B and 5-8F cells, which was reversed in cells subjected to cilengitide treatment or ITGB3 knockout (**Figure 4G**). Small-molecule inhibitors of Akt (AZD5363), Stats (SH-4-54), and Erk1/2 (SCH772984) inhibition assay indicated that SCH772984 treatment, rather than SH-4-54 and AZD5363, abolished the P-EVs- and ITGB3-induced Nrf2 and ATF4 upregulation in 6-10B and 5-8F cells (**Figure 4H and Figure S4**). ChIP-qPCR assay further revealed that both Nrf2 and ATF4 were recruited to the promoter region of the human SLC7A11 gene in 6-10B and 5-8F cells (**Figure 4I**). Overall, these data indicate that P-EVs-upregulated ITGB3 promotes SLC7A11 expression not only by maintaining protein stability but also through the expression of transcription factors upregulated by the MAPK/ERK pathway.

### P-EVs inhibit ferroptosis and promote metastasis of NPC cells by ITGB3-mediated SLC7A11 upregulation

**Figure 5:**
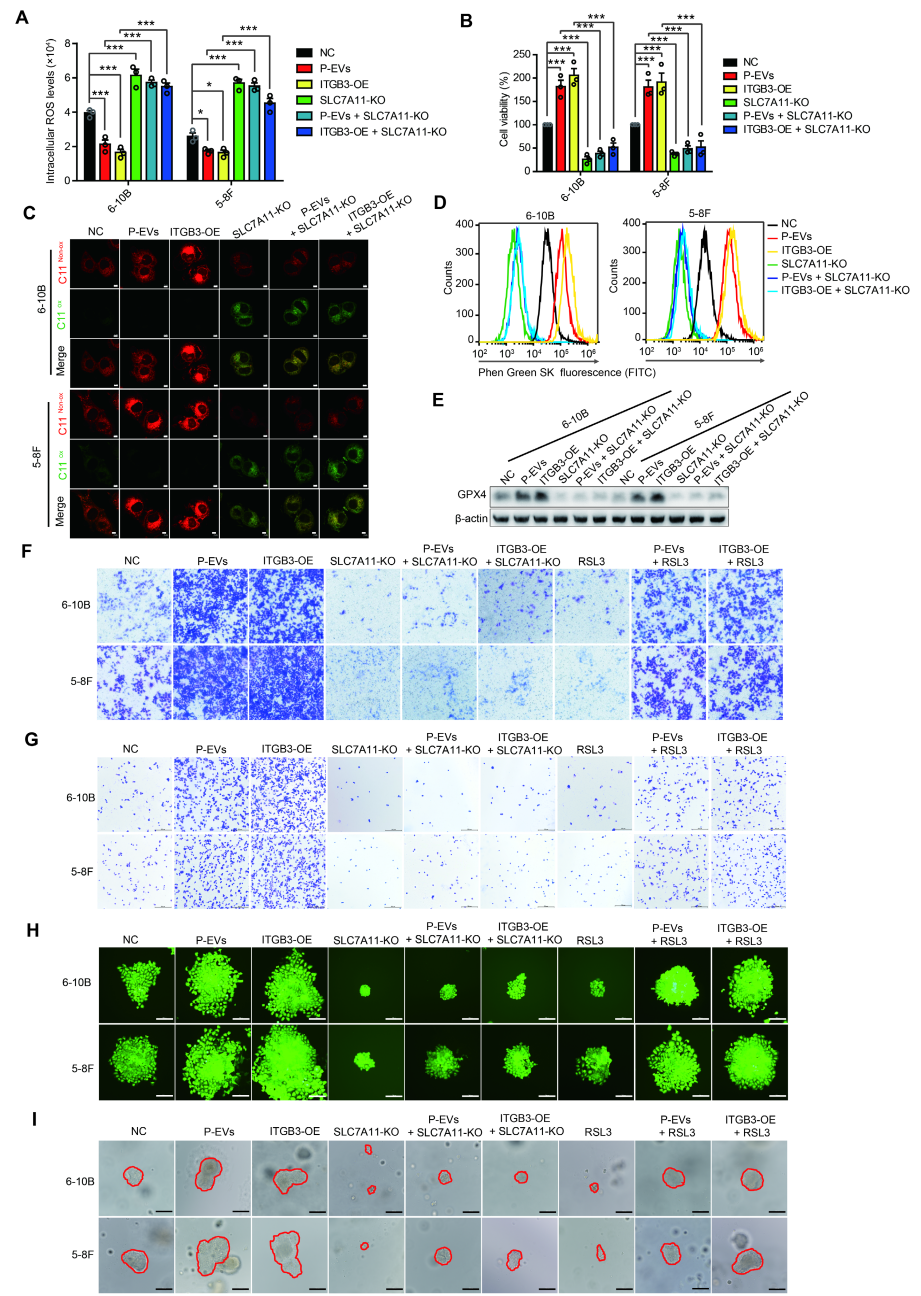
**P-EVs inhibit ferroptosis and promote metastasis of NPC cells through ITGB3-upregulated SLC7A11.** (**A-B**) Intracellular ROS levels (**A**) and cell viability (**B**) of P-EVs-treated, ITGB3-overexpressed, SLC7A11 knockout, SLC7A11 knockout combined with P-EVs treated, and SLC7A11 knockout combined with ITGB3 overexpressed 6-10B and 5-8F cells by fluorescence microplate reader and CCK-8 assay. (**C**) Confocal imaging showed the lipid peroxidation activity (Green) using C11 BODIPY 581/591 in P-EVs-treated, ITGB3-overexpressed, SLC7A11 knockout, SLC7A11 knockout combined with P-EVs treated, and SLC7A11 knockout combined with ITGB3 overexpressed 6-10B and 5-8F cells. C11^Non-ox^ = non-oxidized C11 (Red), C11^Ox^ = oxidized C11 (Green) (Scale bar, 5 µm). (**D**) Flow cytometry analysis of intracellular free iron (Fe^2+^) levels in P-EVs-treated, ITGB3-overexpressed, SLC7A11 knockout, SLC7A11 knockout combined with P-EVs treated, and SLC7A11 knockout combined with ITGB3 overexpressed 6-10B and 5-8F cells using the fluorescent indicator Phen Green SK. (**E**) Western blot analysis of GPX4 expression in P-EVs-treated, ITGB3-overexpressed, SLC7A11 knockout, SLC7A11 knockout combined with P-EVs treated, and SLC7A11 knockout combined with ITGB3 overexpressed 6-10B and 5-8F cells. (**F-I**) Cell invasion (**F**), cell adhesion (**G**), single-cell-derived clone growth (**H**), and tumorsphere propagation (**I**) of P-EVs-treated, ITGB3-overexpressed, SLC7A11 knockout, SLC7A11 knockout combined with P-EVs treated, SLC7A11 knockout combined with ITGB3 overexpressed, RSL3-treated, P-EVs and RSL3-co-treated, and ITGB3 overexpression combined with RSL3 treated 6-10B and 5-8F cells using matrigel invasion assay, adhesion assay, clonogenic assay (Scale bar, 200 µm), and 3D spheroid forming assay (Scale bar, 200 µm), respectively. Experiments were repeated at least thrice. Data represent the mean ± SD (^*^p<.05; ^***^p<.001).

To further investigate the interrelations between ITGB3-upregulated SLC7A11, ferroptosis, and metastasis in NPC cells, we constructed SLC7A11 knockout 6-10B and 5-8F cells (**Figure S5A**). Intracellular ROS detection showed that SLC7A11 knockout inverted P-EVs- and ITGB3-suppressed ROS in 6-10B and 5-8F cells (**Figure 5A**). Similarly, SLC7A11 knockout also reversed P-EVs- and ITGB3-induced GSH/GSSG ratio and -enhanced cell viability in 6-10B and 5-8F cells (**Figure S5B and Figure 5B**). BODIPY™ 581/591 C11 and JC-10 probe labeling demonstrated that SLC7A11 knockout antagonized the P-EVs- and ITGB3-inhibited lipid peroxidation and decrease of mitochondrial membrane potential (**Figure 5C, Figure S5C and S5D**). Meanwhile, SLC7A11 knockout induced upregulation of intracellular iron (Fe^2+^) levels and downregulation of GPX4 expression in 6-10B and 5-8F cells, which restricted the P-EVs- and ITGB3-mediated downregulation of intracellular iron (Fe^2+^) levels and upregulation of GPX4 (**Figure 5D, 5E and Figure S5E**), indicating that P-EVs-upregulated ITGB3 expression inhibits ferroptotic cell death by increasing SLC7A11 expression.

Next, we examined whether SLC7A11 was responsible for metastasis facilitated by P-EVs-mediated upregulation of ITGB3. Wound healing and invasion assays again demonstrated that P-EVs treatment and ITGB3 overexpression dramatically promoted cell migration and invasion in 6-10B and 5-8F cells, which was counteracted by SLC7A11 knockout (**Figure S5F and Figure 5F**). Remarkably, although RSL3 treatment suppressed the migration and invasion of 6-10B and 5-8F cells, P-EVs treatment and ITGB3 overexpression still facilitated the migration and invasion of 6-10B and 5-8F cells in the presence of RSL3 (**Figure S5F and Figure 5F**), suggesting that P-EVs-upregulated ITGB3 expression promoted the metastasis of NPC cells through SLC7A11-suppressed ferroptosis. Subsequent adhesion and clone formation assays also highlighted that SLC7A11 knockout abolished P-EVs- and ITGB3-promoted cell adhesion and colony formation in 6-10B and 5-8F cells. However, P-EVs and ITGB3 restricted the cell adhesion- and colony formation-suppressing effects of RSL3 (**Figure 5G, 5H and Figure S5G**). In parallel, P-EVs- and ITGB3-enhanced MET processes were reversed by SLC7A11 knockout but were maintained in the presence of RSL3 (**Figure S5H**). Most importantly, in 3D spheroid cultures, P-EVs-treated and ITGB3-overexpressed NPC cells formed more single-cell-derived spheres with a larger and more compact morphology, which was almost completely inhibited upon SLC7A11 knockout. However, P-EVs treatment and ITGB3 overexpression significantly antagonized the inhibitory effect of RSL3 on the single cell-derived sphere growth of 6-10B and 5-8F cells (**Figure 5I**). Overall, the data indicate that P-EVs-upregulated ITGB3 expression inhibits ferroptotic cell death by increasing SLC7A11 expression, thereby facilitating metastasis of NPC cells.

### P-EVs-induced ITGB3 upregulation enhances NPC cells growth and distant metastasis in a xenograft model

**Figure 6:**
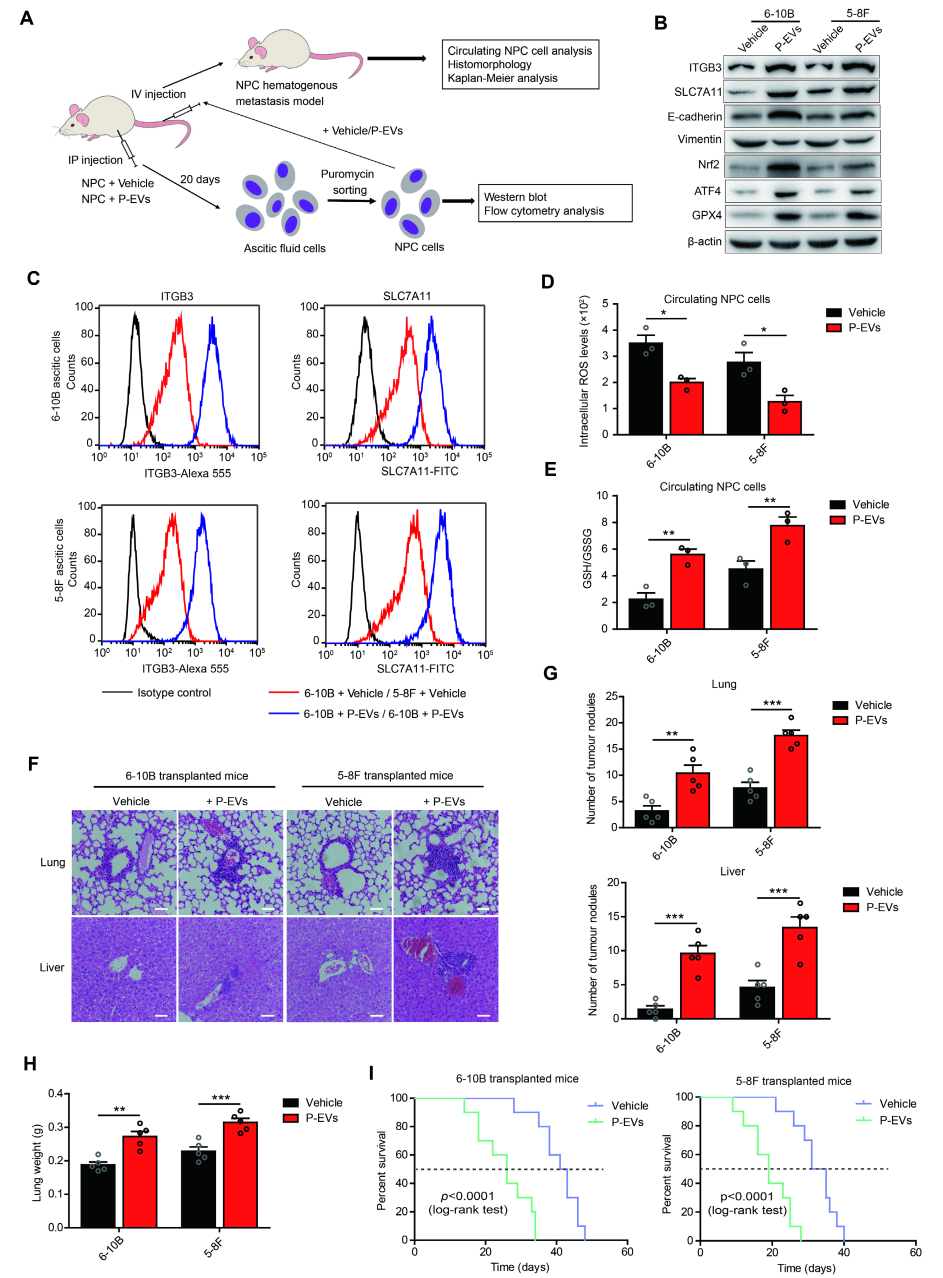
** P-EVs enhance distant metastasis of NPC cells and cause shortened survival of NPC-bearing mice.** (**A**) Experimental design of cell transplantation with 6-10B and 5-8F cells by intraperitoneal (IP) injection and intravenous (IV) injection, respectively, and subsequent *in vivo* studies. (**B**) Western blot analysis of ITGB3, SLC7A11, E-cadherin, Vimentin, Nrf2, ATF4, and GPX4 in 6-10B and 5-8F ascitic cells developed from intraperitoneally injected cells following treatment with or without P-EVs. (**C**) Flow cytometric analysis of cell surface expression of ITGB3 and SLC7A11 on 6-10B and 5-8F ascitic cells developed from intraperitoneally injected cells following treatment with or without P-EVs. Mouse IgG and Rabbit IgG were used as isotype control, respectively. (**D-E**) Intracellular ROS levels (**D**) and cellular GSH/GSSG ratio (**E**) in vehicle- and P-EVs-treated circulating NPC cells by fluorescence microplate reader. (**F**) Histological analysis of the lung and liver tissues obtained from 6-10B- and 5-8F-transplanted mice following treatment with or without P-EVs (Scale bar, 50 µm). (**G**) Number of tumor nodules in the lung and liver metastases of 6-10B and 5-8F hematogenous metastatic mouse model following treatment with or without P-EVs. (**H**) Wet lung weight of 6-10B- and 5-8F-transplanted mice following treatment with or without P-EVs. (**I**) Kaplan-Meier survival curves for mice intravenously transplanted with 6-10B and 5-8F cells following treatment with or without P-EVs. P values were determined by the log-rank (Mantel-Cox) test. Experiments were repeated at least thrice. Data represent the mean ± SD (^**^p<.01; ^***^p<.001).

To further confirm the role of P-EVs in NPC cell ferroptosis and distant metastasis, 6-10B and 5-8F (1×10^6^) cells containing puromycin resistance were intraperitoneally co-injected with P-EVs (10 µg) into BALB/c nude mice to generate ascitic cells, followed by selection using puromycin for *in vitro* detection and an intravenous transplantation mouse model (**Figure 6A**). Consistent with *in vitro* results, P-EVs treatment increased ITGB3 expression and activated the MET program in 6-10B and 5-8F ascitic cells, which was accompanied by elevated expression levels of SLC7A11, Nrf2, ATF4, and GPX4 (**Figure 6B**). Flow cytometry analysis also confirmed that P-EVs-treated ascitic cells 6-10B and 5-8F presented increased levels of ITGB3 and SLC7A11 (**Figure 6C**). Meanwhile, P-EVs treatment downregulated lipid peroxidation in 6-10B and 5-8F ascitic cells (**Figure S6**), indicating that P-EVs induce ITGB3 and SLC7A11 upregulation and ferroptosis inhibition in NPC cell, and therefore play a novel non-redundant facilitating role in NPC cells survival *in vivo*.

Since our data showed that P-EVs promoted NPC metastasis through MET process rather than EMT, we believed that P-EVs mainly promoted NPC distant metastasis through blood circulation. The 6-10B and 5-8F cells were then treated with or without P-EVs (10 µg) and injected intravenously into BALB/c nude mice to generate a hematogenous metastatic mouse model. Circulating NPC cells from peripheral blood analysis showed that P-EVs treatment reduced the intracellular ROS levels and increased the GSH/GSSG ration of NPC cells during blood circulation (**Figure 6D and 6E**). Histopathological results of HE staining illustrated that P-EVs treatment advanced hematogenous metastasis and the formation of secondary metastatic foci of NPC in the lungs and livers of mice bearing 6-10B and 5-8F xenografts, damaging the pulmonary and hepatic architecture, including vascular structures and germinal centers, and an increase in the number of tumor nodules and lung weight (**Figure 6F, 6G, and 6H**). Moreover, P-EVs-treated 6-10B and 5-8F cell-transplanted mice had significantly reduced survival time compared to that of vehicle-treated 6-10B and 5-8F cell-transplanted mice (**Figure 6I**). Taken together, these data, along with the above *in vitro* and *in vivo* results, demonstrate that platelet-secreted EVs from NPC patients can be absorbed by NPC cells and induce increased ITGB3 and SLC7A11 expression to inhibit ferroptosis, and thereby accelerating distant metastasis and the formation of metastatic foci of NPC cells through the MET process.

## Discussion

Distant metastasis is the main cause of death in NPC and is more commonly diagnosed in NPC than in other head and neck squamous cell carcinoma 32. Predominant types of distant metastasis of tumors include lymphatic and hematogenous metastases 33. As one of the main components of human blood, platelets participate in multiple steps of hematogenous metastasis, including helping tumor cells escape immune killing, overcoming the countering effects of shear force, arresting and adhering to the vessel wall, and extravasation 7, 34. However, the role of platelets in the hematogenous metastasis of NPC cells and the pathological mechanism of the interaction between platelets and NPC cells remain unclear and warrant further research. In this study, we found that platelet-derived EVs from NPC patients can be taken up by NPC cells, thereby promoting NPC cell migration, invasion, adhesion, and clone formation by upregulating ITGB3 expression. This is accompanied by increased MET process (**Figure 1 and Figure 2**), suggesting that P-EVs advance distant metastasis through blood circulation and induce the formation of metastatic foci in distant organs of NPC. To confirm this conclusion, we established a hematogenous metastatic mouse model through tail vein injection of NPC cells in BALB/c nude mice and found that P-EVs treatment led to a decrease of oxidative stress levels in circulating NPC cells and increased the number of tumor nodules in the lungs and liver (**Figure 6**). These data elucidate the molecular mechanism by which platelets promote the distant metastasis of NPC and demonstrate that P-EVs could be clinically significant for the diagnosis and treatment of NPC.

When tumor cells metastasize through the bloodstream, they activate platelets and stimulate platelet aggregation 35. Activated platelets, in turn, mediate the growth and metastasis of circulating tumor cells, and therefore, form a positive feedback loop 11, 35. Mouse models and clinical studies have also shown that thrombocytosis promotes the metastasis of tumor cells *in vivo* and is related to the poor prognosis of patients with tumor 36, 37. In this study, we first revealed the role of platelets in promoting the distant metastasis of NPC through EVs. Interestingly, we demonstrated that EVs derived from platelets of healthy volunteers did not show an evident promotive effect compared to EVs derived from platelets of NPC patients (**Figure 1**), implying that platelet-derived EVs from healthy people and NPC patients have different physiological and pathological effects, which may be due to differences in secretion patterns and contents of EVs in healthy people and patients with NPC. Current research shows that tumor cells can increase thrombin expression by either upregulating the tissue factors or directly secreting thrombin to activate platelets 38-40. Although thrombin was used to activate healthy human platelets and isolated EVs, EVs derived from thrombin-activated healthy human platelets still had no evident tumor metastasis-promoting effect compared to thrombin-activated NPC patient platelet-derived EVs. This suggests that just as tumor cells secrete IgG differently from normal IgG to activate platelets 41, other platelet activation pathways may already exist in NPC patients to mediate the secretion of EVs. Further studies of tumor cell-induced platelet activation and EVs secretion patterns in NPC patients may be helpful in revealing the mechanism of platelet-promoting tumor metastasis through EVs.

Notably, ITGB3 was elevated in platelets and platelet-derived EVs of NPC patients, but not in platelets and platelet-derived EVs isolated from healthy volunteers. ITGB3 was found to play a promoting role in the metastasis of NPC cells and the levels of ITGB3 expression was positively correlated with distant metastasis of NPC patients. Different proteins in EVs often determine the organ targeting of tumor metastasis by preparing the premetastatic niche or modulating the expression of specific proteins in tumor cells 16, 21. The data indicated that P-EVs-absorbed NPC cell-transplanted mice presented increased numbers of tumor nodules in the lungs and liver by upregulating ITGB3, suggesting that P-EVs-induced ITGB3 upregulation likely mediate lung and liver metastasis in NPC.

EMT is a key pathological transformation procedure that converts stationary epithelial tumor cells into motile mesenchymal cells during the initiation of metastasis 24. During this process, tumor cells lose their adherent junction characteristics and acquire the ability to migrate and invade, leading to the dissemination of single tumor cells 24, 42. With the metastasis of tumor cells in the lymphatics and bloodstream, metastasized cancer cells undergo MET and regain the epithelial phenotype to permit their settlement and ensure the formation of secondary metastatic foci at distant sites 43, 44. Our findings demonstrate that P-EVs treatment promotes the metastasis of NPC cells while enhancing focal adhesion and single-cell clonogenic ability by upregulating ITGB3, which is accompanied by MET induction (**Figure 2**). This indicates that as one of the main components involved in hemostasis and thrombosis, platelets mediate distant metastasis and the formation of secondary metastatic foci at distant organs of NPC through a P-EVs-upregulated ITGB3 initiated MET program.

Ferroptosis is an iron-dependent form of cell death driven by the accumulation of lipid-ROS and reduction of GSH 45. Circulating cancer cells in the blood experience high levels of oxidative stress, including increased ROS levels and decreased GSH/GSSG ratio, thereby inhibiting the distant metastasis of these cancer cells 28. In the current study, we found that P-EVs treatment led to a decrease in intracellular ROS, lipid-ROS, mitochondrial damage, intracellular iron (Fe^2+^) and an increase in the GSH/GSSG ratio, that is, suppressed ferroptosis, by upregulating ITGB3 and therefore enhancing the distant metastasis of NPC cells (**Figure 3 and Figure 5**). This suggests that in the process of hematogenous metastasis of NPC cells in NPC patients, platelets inhibit oxidative damage and ferroptosis-related signatures through EVs-upregulated ITGB3, which ensures the survival of NPC cells in distant metastasis.

In summary, this study revealed that EVs derived from platelets of NPC patients inhibit ferroptosis by upregulating ITGB3, thereby promoting the distant metastasis of NPC cell and accelerating the formation of secondary metastatic foci at distant organs of NPC. These findings not only unveil the pathological mechanism of platelets in NPC metastasis, but also elucidate the mechanism of platelet-tumor cell interactions mediated by EVs. As platelets are one of the most common components in the blood and mediate distant metastasis of cancer cells, these findings could potentially assist the study of other tumors and may be helpful in promoting the application of platelet-targeted drugs in patients with tumor.

## Supplementary Material

Supplementary figures, materials and methods, and table.

## Abbreviations

NPC: Nasopharyngeal carcinoma
EVs: Extracellular vesicles
ITGB3: Integrin β3
DIC: Differential interference contrast
KO: knock-out
NTA: Nanoparticle tracking analysis
Co-IP: Co-immunoprecipitation
ChIP-qPCR: Chromatin immunoprecipitation and quantitative PCR
EMT: Epithelial-mesenchymal transition
MET: Mesenchymal-epithelial transition
ROS: Reactive oxygen species
BSO: Buthionine sulphoximine
