# Supplementary material for: Platelet-derived extracellular vesicles inhibit ferroptosis and promote distant metastasis of nasopharyngeal carcinoma by upregulating ITGB3
Source: Int J Biol Sci. 2022 Sep 24;18(15):5858–72. doi: 10.7150/ijbs.76162 (PMC9576525; doi:10.7150/ijbs.76162)
Supplement: Supplementary file 1 — Supplementary figures, materials and methods, and table. [file ijbsv18p5858s1.pdf]

Supplementary Materials

Platelet-derived extracellular vesicles inhibit ferroptosis and promote distant metastasis of nasopharyngeal carcinoma by upregulating ITGB3

Fei Li, Ting Xu, Peiling Chen, Rui Sun, Chaoyi Li, Xin Zhao, Jinxin Ou, Jingyao Li, Taoshu Liu, Maozhen Zeng, Weizhong Zheng, Yunchen Lin, Le Yang, Zecang Li, Haisheng Chen, Qing Zhang

Supplementary Figures and Tables

Figure S1
Figure S2
Figure S3
Figure S4
Figure S5
Figure S6
Table S1

Supplemental Materials and Methods

Supplemental Figures

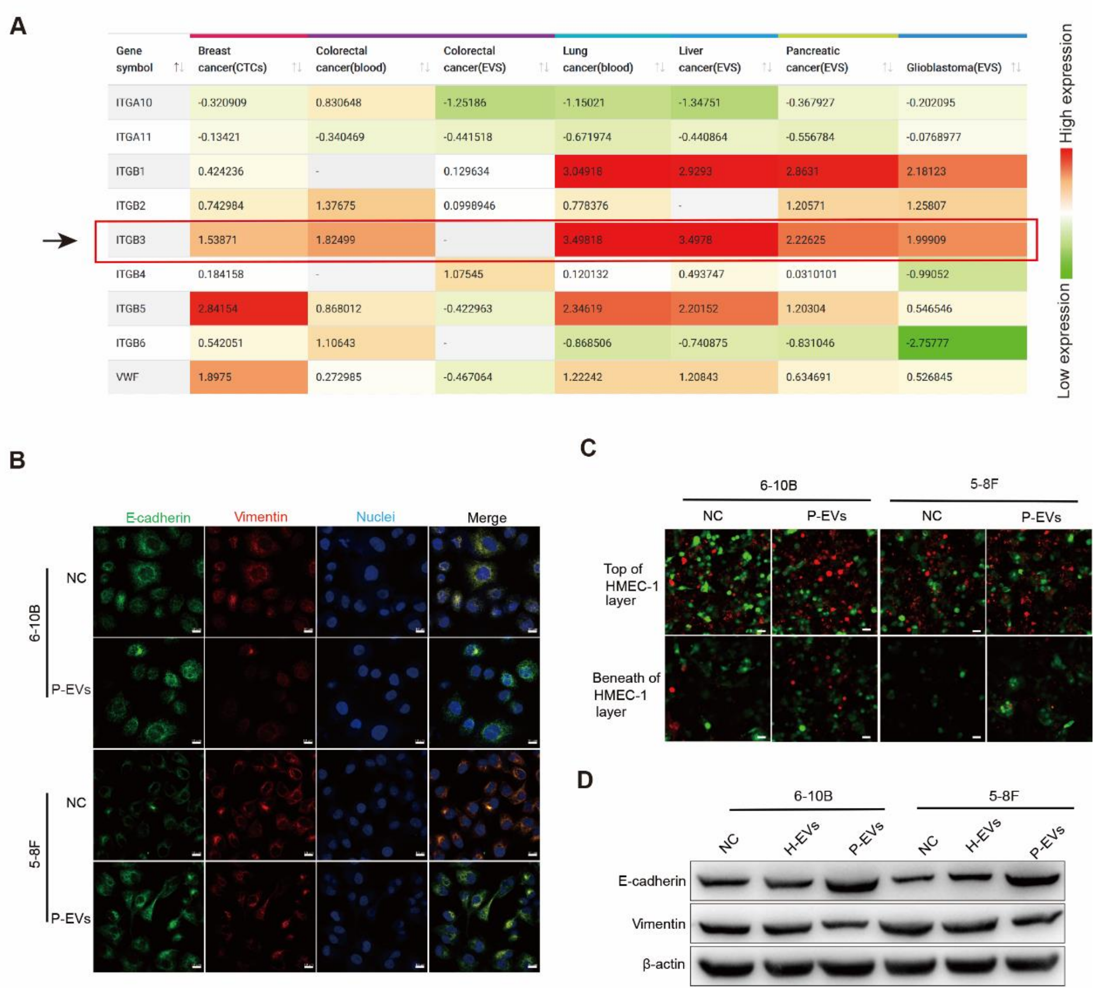

Gene symbol	Breast cancer(CTCs)	Colorectal cancer(blood)	Colorectal cancer(EVS)	Lung cancer(blood)	Liver cancer(EVS)	Pancreatic cancer(EVS)	Glioblastoma(EVS)
ITGA10	-0.320909	0.830648	-1.25186	-1.15021	-1.34751	-0.367927	-0.202095
ITGA11	-0.13421	-0.340469	-0.441518	-0.671974	-0.440864	-0.556784	-0.0768977
ITGB1	0.424236	-	0.129634	3.04918	2.9293	2.8631	2.18123
ITGB2	0.742984	1.37675	0.0998946	0.778376	-	1.20571	1.25807
ITGB3	1.53871	1.82499	-	3.49818	3.4978	2.22625	1.99909
ITGB4	0.184158	-	1.07545	0.120132	0.493747	0.0310101	-0.99052
ITGB5	2.84154	0.868012	-0.422963	2.34619	2.20152	1.20304	0.546546
ITGB6	0.542051	1.10643	-	-0.868506	-0.740875	-0.831046	-2.75777
VWF	1.8975	0.272985	-0.467064	1.22242	1.20843	0.634691	0.526845

Figure S1. P-EVs promote metastasis of NPC cells

(**A**) BBCancer database analysis of ITGB3 expression in multiple tumor blood samples, extracellular vesicles (EVs), and circulating tumor cells (CTCs). (**B**) Immunofluorescence microscopy of E-cadherin and Vimentin in 6-10B and 5-8F pretreated with P-EVs. Hoechst 33342 was used to stain the nuclei of cells (blue) (Scale bar, 10 μm). (**C**) Representative z-stack images of HMEC-1 cells (red) plated on glass bottom dishes. P-EVs-treated or untreated 6-10B-GFP and 5-8F-GFP cells were gently overlaid onto the endothelial cell layer and incubated for 2 h. NPC cells transmigration across the endothelial layer were scored by confocal imaging (Scale bar, 20 μm). (**D**)

Western blot analysis for E-cadherin and Vimentin in H-EVs-treated, P-EVs-treated, and -untreated 6-10B and 5-8F cells released from 3D spheroid culture. Experiments were performed in triplicate.

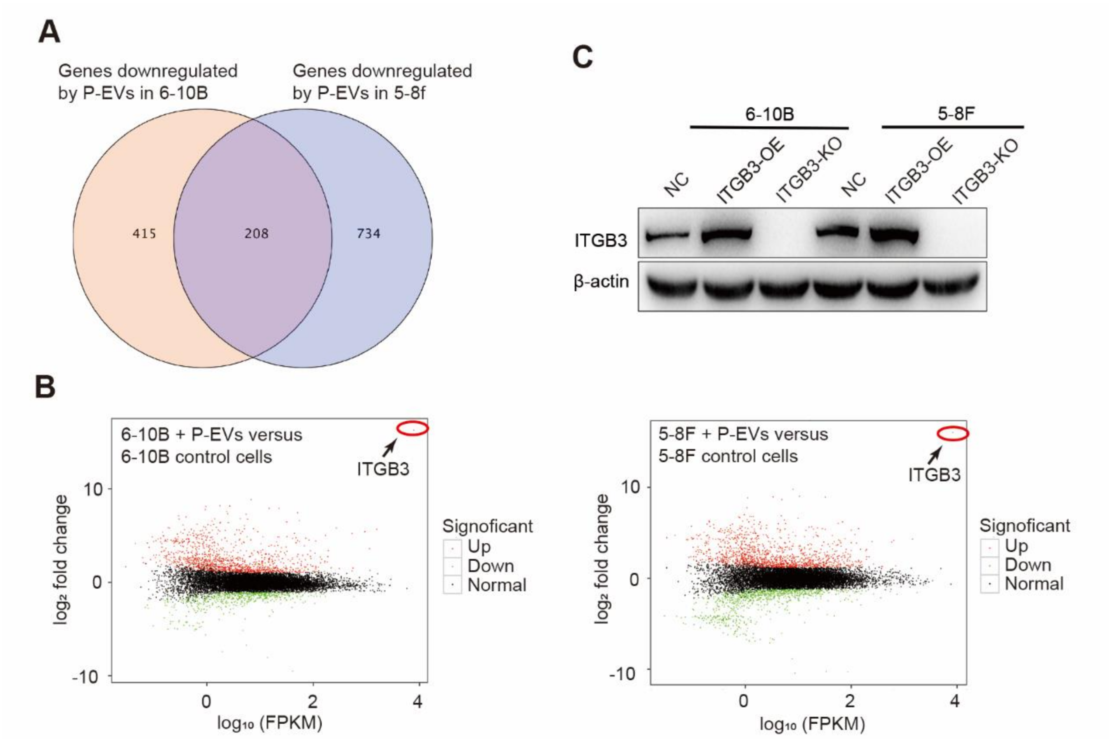

Figure S2. P-EVs upregulate ITGB3 expression in NPC cells

(**A**) Expression of potential genes downregulated by P-EVs treatment in 6-10B and 5-8F cells; numbers indicate quantity of genes in each differentially expressed genes (DEGs) subset. (**B**) MA plot for analyzing DEGs comparing 6-10B and 5-8F control cells and P-EVs-treated 6-10B and 5-8F cells. (**C**) Western blot analysis of ITGB3 expression in ITGB3-overexoressed and ITGB3 knockout 6-10B and 5-8F cells. Experiments were performed in triplicate.

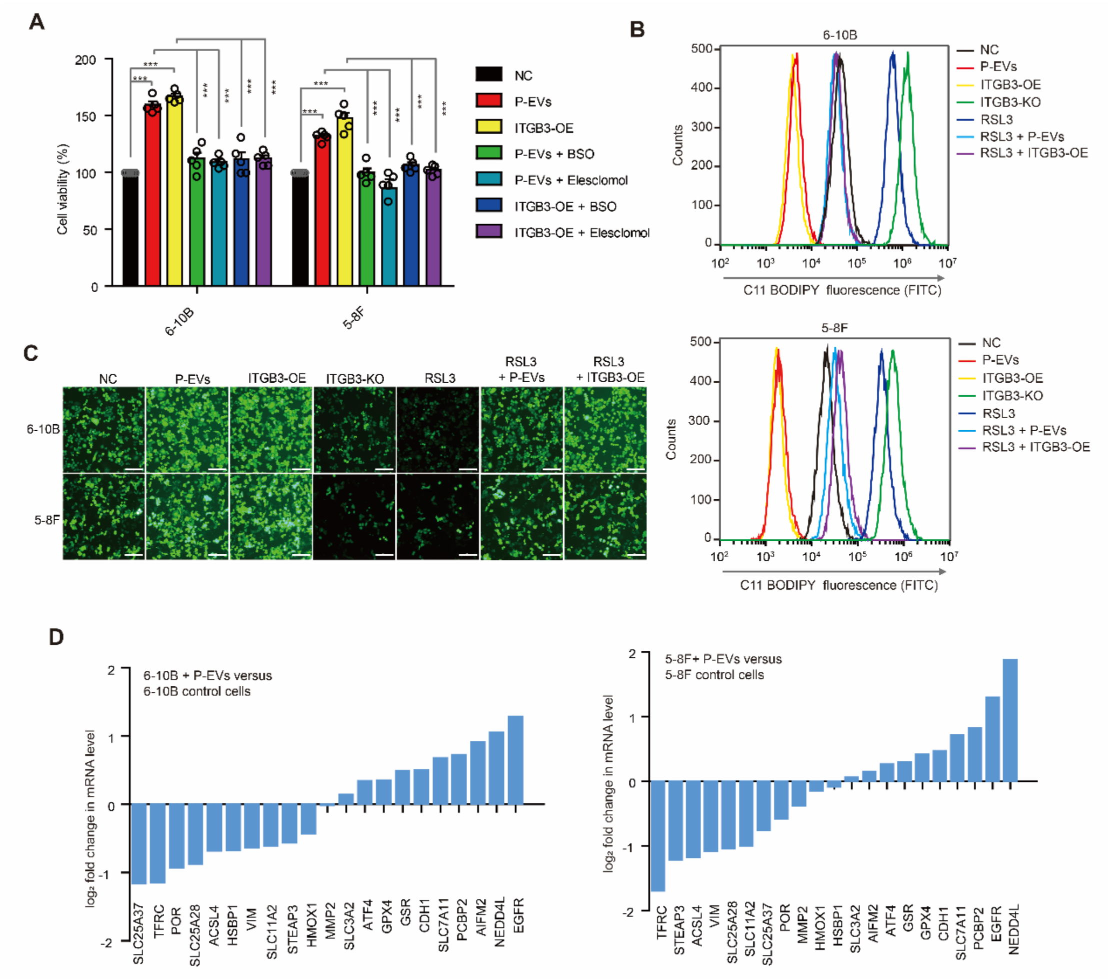

Figure S3. P-EVs inhibit ferroptosis in NPC cells by upregulating ITGB3

(**A**) Cell viability of P-EVs-treated, ITGB3-overexpressed, P-EVs and BSO (10 µM)-co-treated, P-EVs and elesclomol (10 µM) co-treated, ITGB3 overexpression combined with BSO treated, and ITGB3 overexpression combined with elesclomol treated 6-10B and 5-8F cells by the CCK-8 assay. (**B**) Flow cytometry analysis of lipid peroxidation activity using C11 BODIPY 581/591 in P-EVs-treated, ITGB3-overexpressed, ITGB3 knockout, RSL3 (10 µM)-treated, RSL3 and P-EVs-co-treated, and ITGB3 overexpression combined with RSL3 treated 6-10B and 5-8F cells. (**C**) Intracellular free iron (Fe^{2+}) levels in P-EVs-treated, ITGB3 overexpressed, ITGB3 knockout, RSL3-treated, RSL3 and P-EVs co-treated, and ITGB3 overexpression combined with

RSL3 treated 6-10B and 5-8F cells, using the fluorescent indicator Phen Green SK (green) (Scale bar, 200 μm). (**D**) Column diagram represents the expression of genes correlated with ferroptosis with P-EVs treatment in 6-10B and 5-8F cells. Experiments were repeated at least thrice. Data represent the mean ± SD ($^{***}p<.001$).

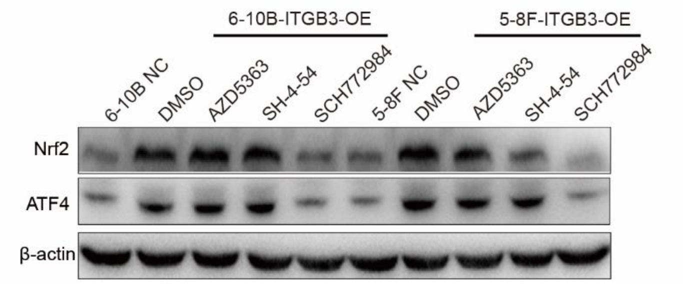

Figure S4. Western blot of Nrf2 and ATF4 in ITGB3 overexpressed 6-10B and 5-8F cells pretreated with DMSO control, AZD5363, SH-4-54, and SCH772984

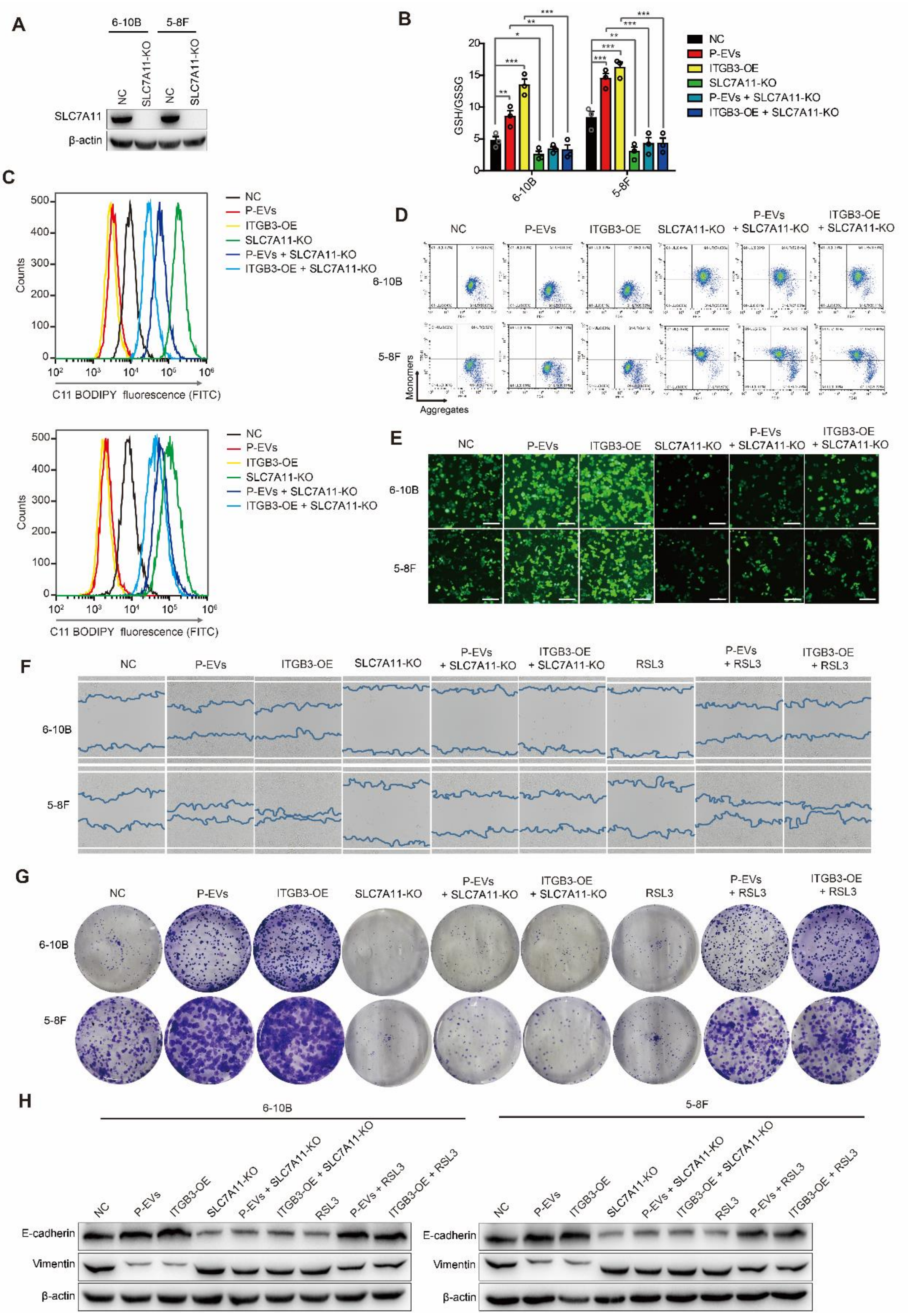

Figure S5. P-EVs inhibit ferroptosis and promote metastasis of NPC cells through ITGB3-upregulated SLC7A11

(**A**) Western blot of SLC7A11 expression in wildtype and SLC7A11 knockout 6-10B

and 5-8F cells. (**B**) Cellular GSH/GSSG ratio in P-EVs-treated, ITGB3-overexpressed, SLC7A11 knockout, SLC7A11 knockout combined with P-EVs treated, and SLC7A11 knockout combined with ITGB3 overexpressed 6-10B and 5-8F cells by fluorometric microplate format. (**C**) Flow cytometry analysis of lipid peroxidation activity using C11 BODIPY 581/591 in P-EVs-treated, ITGB3-overexpressed, SLC7A11 knockout, SLC7A11 knockout combined with P-EVs treated, and SLC7A11 knockout combined with ITGB3 overexpressed 6-10B and 5-8F cells. (**D**) Flow cytometry analysis of mitochondrial membrane potential in P-EVs-treated, ITGB3-overexpressed, SLC7A11 knockout, SLC7A11 knockout combined with P-EVs treated, and SLC7A11 knockout combined with ITGB3 overexpressed 6-10B and 5-8F cells using JC-10. (**E**) Intracellular free iron (Fe^{2+}) levels in P-EVs-treated, ITGB3-overexpressed, SLC7A11 knockout, SLC7A11 knockout combined with P-EVs treated, and SLC7A11 knockout combined with ITGB3 overexpressed 6-10B and 5-8F cells using the fluorescent indicator Phen Green SK (green) (Scale bar, 200 μm). (**F**) Wound healing assay showing cell migration of P-EVs-treated, ITGB3-overexpressed, SLC7A11 knockout, SLC7A11 knockout combined with P-EVs treated, SLC7A11 knockout comb ined with ITGB3 overexpressed, RSL3-treated, P-EVs and RSL3-co-treated, and ITGB3 overexpression combined with RSL3 treated 6-10B and 5-8F cells. (**G**) Clone formation capacity of P-EVs-treated, ITGB3-overexpressed, SLC7A11 knockout, SLC7A11 knockout combined with P-EVs treated, SLC7A11 knockout combined with ITGB3 overexpressed, RSL3-treated, P-EVs and RSL3-co-treated, and ITGB3 overexpression combined with RSL3 treated 6-10B and 5-8F cells using clonogenic assays. (**H**)

Western blot analysis for E-cadherin and Vimentin in P-EVs-treated, ITGB3-overexpressed, SLC7A11 knockout, SLC7A11 knockout combined with P-EVs treated, SLC7A11 knockout combined with ITGB3 overexpressed, RSL3-treated, P-EVs and RSL3-co-treated, and ITGB3 overexpression combined with RSL3 treated 6-10B and 5-8F cells. Experiments were repeated at least thrice. Data represent the mean ± SD ($^{*}p<.05$; $^{**}p<.01$; $^{***}p<.001$).

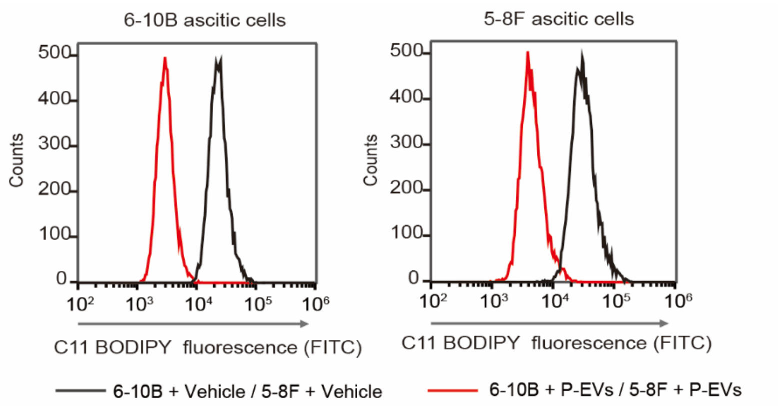

Figure S6. Flow cytometry analysis of lipid peroxidation activity using C11 BODIPY 581/591 in 6-10B and 5-8F ascitic cells developed from intraperitoneally injected cells following treatment with or without P-EVs.

Table S1. Information of antibodies used in this study

Antibody	Cat.No.	Clonality	Host	Manufacturer	Town	Country	Applications /Dilutions
anti-ITGB3	ab179473	Monoclonal	Rabbit	Abcam	Cambridge	UK	WB/1:1000<br>IP/1:40
anti-ITGB3	NBP2-53414	Monoclonal	Mouse	Novus	Colorado	USA	Flow Cyt/1μg/ml<br>IF/1μg/ml
anti- SLC7A11	ab175186	Monoclonal	Rabbit	Abcam	Cambridge	UK	WB/1:1000<br>IP/1:50
anti- SLC7A11	NB300-318SS	Monoclonal	Rabbit	Novus	Colorado	USA	Flow Cyt/2μg/ml<br>IF/1:100
anti-E-cadherin	ab40772	Monoclonal	Rabbit	Abcam	Cambridge	UK	WB/1:10000<br>IF/1:500
anti-Vimentin	ab20346	Monoclonal	Mouse	Abcam	Cambridge	UK	WB/1:1000<br>IF/1μg/ml
anti-CD63	ab134045	Monoclonal	Rabbit	Abcam	Cambridge	UK	WB/1:1000
anti-CD9	ab236630	Monoclonal	Rabbit	Abcam	Cambridge	UK	WB/1:1000
anti-ALIX	ab186728	Monoclonal	Rabbit	Abcam	Cambridge	UK	WB/1:1000
anti-TSG-101	ab125011	Monoclonal	Rabbit	Abcam	Cambridge	UK	WB/1:1000
anti-AKT	ab179463	Monoclonal	Rabbit	Abcam	Cambridge	UK	WB/1:10000
anti-P-AKT	ab192623	Monoclonal	Rabbit	Abcam	Cambridge	UK	WB/1:1000
anti-STAT3	ab68153	Monoclonal	Rabbit	Abcam	Cambridge	UK	WB/1:1000
anti-P-STAT3	ab32143	Monoclonal	Rabbit	Abcam	Cambridge	UK	WB/1:2000
anti-ERK1/2	ab184699	Monoclonal	Rabbit	Abcam	Cambridge	UK	WB/1:10000
anti-P-ERK1/2	ab223500	Monoclonal	Rabbit	Abcam	Cambridge	UK	WB/1:400
anti-GPX4	ab125066	Monoclonal	Rabbit	Abcam	Cambridge	UK	WB/1:1000
anti-Nrf2	ab62352	Monoclonal	Rabbit	Abcam	Cambridge	UK	WB/1:1000<br>CHIP/2μg/25μg
anti-ATF4	ab85049	Monoclonal	Rabbit	Abcam	Cambridge	UK	WB/1:1000<br>CHIP/2μg/25μg
anti-ubiquitin	#3936	Monoclonal	Mouse	Cell Signaling Technology	Massachusetts	USA	WB/1:1000
anti-β-actin	ab115777	Monoclonal	Rabbit	Abcam	Cambridge	UK	WB/1:200
anti-GAPDH	ab8245	Monoclonal	Mouse	Abcam	Cambridge	UK	WB/1:1000
Rabbit IgG	ab172730	Monoclonal	Rabbit	Abcam	Cambridge	UK	IP/1:50<br>CHIP /2μg/25μg

							Flow Cyt/2μg/ml
Mouse IgG	ab 37355	Monoclonal	Mouse	Abcam	Cambridge	UK	Flow Cyt/1μg/ml
FITC-labeled goat anti-rabbit IgG	ab6717	/	Goat	Abcam	Cambridge	UK	Flow Cyt/1:1000 IF/1:1000
Alexa 555-labeled goat anti-mouse IgG	Ab150114	/	Goat	Abcam	Cambridge	UK	Flow Cyt/1:2000 IF/1:1000
HRP-labeled goat anti-rabbit IgG	7074S	/	Goat	Cell Signaling Technology	Massachusetts	USA	WB/1:3000
HRP-labeled goat anti-mouse IgG	7076S	/	Goat	Cell Signaling Technology	Massachusetts	USA	WB/1:3000

Supplemental materials and methods

Generation of ITGB3 and SLC7A11 knockout cells

Both ITGB3 and SLC7A11 knockout (KO) 6-10B and 5-8F cells were generated using the CRISPR/Cas9 system, as described previously [1]. Briefly, guide RNA (gRNA) targeting ITGB3 or SLC7A11 was constructed into the lentiCRISPR v2 plasmid. 6-10B and 5-8F cells were infected with lentiviruses obtained from HEK293T cells co-transfected with lentiCRISPR v2 coding for the respective sgRNA, psPAX2, and pMD2.G. After selection with 1 μg/ml puromycin for 3 days, single cells were seeded into 96-well plates and cultured for 3 weeks, followed by harvesting for ITGB3 and SLC7A11 KO testing using western blot analysis. The gRNA sequences targeting ITGB3 and SLC7A11 used in the study have been published previously [2, 3].

Western blot and co-immunoprecipitation (Co-IP)

Total proteins were extracted from NPC cells and boiled in RIPA buffer (Abcam, USA) with protease inhibitors. Proteins were separated by sodium dodecyl sulfate–polyacrylamide electrophoresis gel electrophoresis (SDS-PAGE), electrophoretically transferred to polyvinylidene fluoride membranes (Millipore, Germany), and probed with anti-ITGB3, anti-ALIX, anti-TSG101, anti-CD9, anti-CD63, anti-E-cadherin, anti-vimentin, anti-GPX4, anti-SLC7A11, anti-Nrf2, anti-ATF4, anti-phospho-STAT3, anti-STAT3, anti-phospho-ERK1/2, anti-ERK1/2, anti-phospho-AKT, anti-AKT, anti-GAPDH, and anti-β-actin overnight at 4 °C and followed by horseradish peroxidase (HRP)-conjugated goat anti-rabbit IgG or HRP-conjugated goat anti-mouse IgG

incubation at room temperature for 1h. ECL chemiluminescence reagent (Millipore) was applied to determine protein expression levels. Detailed information on the antibodies is summarized in **Table S1**. For Co-IP, whole-cell lysates were incubated with anti-ITGB3 antibodies for 6 h and followed by capture with protein A/G-magnetic beads for 16 h. The bound proteins were then washed, eluted, separated by SDS-PAGE, and detected by western blotting.

Nanoparticle tracking analysis (NTA)

NTA was performed using NanoSight LM10 (NanoSight) as previously described [1]. Briefly, the purified EVs were diluted in PBS solution to achieve a concentration range of 10^7-10^8 particles per ml. Data were obtained by reading three 1-min videos with parameters set at a camera level of 12 and a detection threshold of 3. The average particle size distribution and concentration were analyzed using NTA software (version 3.2 Build 16) and graphs were plotted using GraphPad Prism 5.

Immunofluorescence assay

Immunofluorescence assays were performed as described previously [4]. Briefly, NPC cells were grown on μ-Dish (Ibidi, Germany). After P-EVs (5 μg/ml) treatment, cells were fixed with 4% paraformaldehyde, permeabilized in 0.1% Triton X-100, blocked in 5% BSA-PBS solution, and incubated with primary antibody at 4°C overnight and then corresponding FITC- and Alexa 555-conjugated IgG secondary antibody at 37°C for 1 h. Cell nuclei were stained with Hoechst 33342, and imaged with a 63× objective

using an LSM 880 confocal microscope (Zeiss).

Cell migration and invasion assay

Cell migration and invasion assays were performed as described previously [1]. Briefly, Culture-Insert (Ibidi) was used to perform a wound healing assay to measure cell migration. Culture-Insert was removed to form scratches after NPC cells were seeded onto 24-well plates for 12 h, and the first image of the scratch was recorded. The second image was acquired with a 20 × objective using a Nikon Eclipse Ti2-E microscope (Nikon) after H-EVs (5 μg/ml) or P-EVs (5 μg/ml) treatment for 24 h. For the invasion assay, 1×10^5 NPC cells were seeded into the Matrigel (200 μg/ml)-coated upper chamber of a Transwell chamber (Corning) and co-incubated with H-EVs (5 μg/ml) or P-EVs (5 μg/ml) for 24 h. After non-invading cells were removed with a cotton swab, the cells on the lower membrane surface were fixed in 4% paraformaldehyde and stained with 0.1% crystal violet for imaging.

Clone formation assay

GFP-labeled NPC cells were transferred into 24-well plates at a density of 400 cells/well. Complete medium (500 µL) was replaced every 48 h until cell clones could be observed directly. The size of single clones was recorded by a Nikon EclipseTi2-E fluorescence microscope (Nikon) GFP channel. The clones were fixed in 4% paraformaldehyde and stained with 0.1% crystal violet for counting.

Adhesion assay

EVs-treated NPC cells (1×10^4 cells/well) were seeded in fibronectin (10 μg/ml)-coated 24-well plates (Corning) and incubated for 15 min. After non-adherent cells were washed away, the adherent cells were fixed in 4% paraformaldehyde and stained with 0.1% crystal violet for counting under a Nikon EclipseTi2-E fluorescence microscope (Nikon). For the parallel plate flow adhesion assay, GFP-transfected NPC cells (suspended at 5×10^6/ml in complete medium) were perfused through a fibronectin (10 μg/ml)-coated parallel plate flow chamber (Ibidi) at a shear stress of 5 dynes/cm^2 for 30 min. The chamber was then perfused with fresh complete media for an additional 2 min at 5 dynes/cm^2 to remove the non-adherent cells, and the adherent cells were observed using a Nikon EclipseTi2-E fluorescence microscope (Nikon).

Transendothelial migration assay

The transendothelial migration assay was performed as previously reported[5]. Briefly, mCherry-transfected HMEC-1 cells were seeded into a μ-Dish (Ibidi) to achieve a monolayer of cells. P-EVs (5 μg/ml)-treated 6-10B-GFP and 5-8F-GFP stable cells were then overlaid onto the endothelial layer and incubated for 2 h. Cells were fixed in 4% paraformaldehyde, and z-stack confocal images were acquired from top to bottom of the HMEC-1 cell layer.

3D spheroid cultures

NPC cells were resuspended in RPMI-1640 medium containing 30% FBS at a

concentration of 1×10^6 cells/ml. Cells were mixed with VitroGel Hydrogel Matrix (TheWell, Bioscence, USA) at a 1:2 ratio and 300 µL of the hydrogel/cell mixture was pipetted into a 24-well plate and incubated for 15 min at room temperature. Complete culture medium (300 µL) was added to the top of the hydrogel, and 3D cell cultures were maintained for 10 days. Spheroids were recovered from the hydrogel using VitroGel Cell Recovery Solution (TheWell, Bioscience) according to the manufacturer's instructions for subsequent western blotting and immunofluorescence analysis.

Total RNA isolation and expression analysis

The two cell types (6-10B and 5-8F) were treated with or without P-EVs (5 µg/ml) for 48 h. Total RNA was extracted using TRIzol reagent (Invitrogen, USA) according to the manufacturer's instructions. RNA-seq libraries were prepared, sequenced, and analyzed on an Illumina HiSeq 2500 platform by the Beijing Biomarker Technology Corporation, as described in our previous report [1]. All raw data have been deposited under the Gene Expression Omnibus accession number GSE196879.

Flow cytometry

For flow cytometry analysis of cell surface ITGB3 and SLC7A11, 100 µL NPC cells (1×10^6) were digested into a single-cell suspension and incubated with primary antibody followed by corresponding FITC- and Alexa 555-conjugated IgG secondary antibody. Rabbit IgG or mouse IgG was used as isotype control. Cells were then washed

3 times by centrifugation at 400 g and analyzed using a CytoFLEX flow cytometer (Beckman, Germany).

Intracellular ROS detection

Intracellular ROS levels were measured as previously described [4]. Briefly, NPC cells were incubated with a DCFH-DA probe at 1:1000 dilution in serum-free RPMI medium at 37°C for 30 min, washed with serum-free medium in the dark, and detected by a fluorescent reader (TECAN) at an excitation wavelength of 488 nm.

GSH/GSSG detection

NPC cells were lysed by repeated freeze-thaw cycles and then centrifuged to collect the supernatant. Intracellular GSH and GSSG concentrations were detected using GSH and GSSG Assay Kit (Abbkine), according to the manufacturer's instructions.

Cell viability assay

Growth factor-starved NPC cells were seeded into 96-well plates at a density of 3×10^3 cells/well and treated with P-EVs (5 µg/ml) or various inhibitors or activators for 24 h. Subsequently, the cells were incubated with 10 µL of CCK-8 (Dojindo, Japan) solution for 3 h. Absorbance was measured at 450 nm using a microplate reader (TECAN, Germany). The results are presented as percentages of the control values obtained based on the absorbance of the untreated cells.

Lipid peroxidation assay

NPC cells were seeded in μ-Dish (Ibidi) and treated with P-EVs (5 μg/mL) or RSL3 (10 μM) for 24 h. The cells were incubated with 2 μM BODIPY^{TM} 581/591 C11 (Thermo) at 37 °C for 20 min, washed with Hanks' balanced salt solution, and imaged using LSM 880 confocal microscope (Zeiss) or examined using CytoFLEX flow cytometer (Beckman).

Transmission electron microscopy

Transmission electron microscopy assay was performed as previously described [6]. In brief, NPC cells were collected after P-EVs (5 μg/ml) or RSL3 (10 μM) treatment for 24 h and re-suspended in 2.5% glutaraldehyde (Sigma) at 4 °C for preservation. Fixed cells were washed in 0.1 M PB (pH 7.4) and pre-embedded in 1% agarose solution. Samples were then fixed with 1% OsO_4 and dehydrated by concentration gradient of ethanol. Next, samples were penetrated with resin and kept in 37 °C oven overnight. Resin blocks were then cut to 60 nm section and fished out to 150 meshed cuprum grids and stained with 2 % uranium acetate and 2.6 % lead citrate solution for observation using JEM-1400Flash transmission electron microscopy (JEOL, Japan).

Mitochondrial membrane potential assay

Mitochondrial membrane potential was measured using the JC-10 dye solution (Solarbio, China) as previously described [7]. Briefly, NPC cells were seeded in 6-well plates and treated with P-EVs (5 μg/ml) or RSL3 (10 μM) for 24 h. The cells were

trypsinized and centrifuged at 600 g for 3 min. The pellet was resuspended in 500 µl RPMI-1640 medium and 500 µl JC-10 dye solution at a density of 2 × 10^5 cells/tube and incubated at 37 ºC in 5 % CO_2 for 20 min (in the dark). The membrane potential was analyzed with a CytoFLEX flow cytometer (Beckman) by measuring the fluorescence with excitation at 488 nm and emission at 530 nm and 585 nm.

Intracellular iron (Fe^{2+}) measurement

Intracellular iron (Fe^{2+}) levels were measured using the fluorescent indicator Phen Green SK (Invitrogen) as previously described [8]. Briefly, NPC cells were seeded in 6-well plates and treated with P-EVs (5 µg/ml) or RSL3 (10 µM) for 24 h. The cells were then incubated with 10 µM Phen Green SK in PBS at 37℃ for 10 min, washed with PBS followed by visualized using Nikon EclipseTi2-E fluorescence microscope (Nikon) or analyzed on a CytoFLEX flow cytometer (Beckman).

Chromatin immunoprecipitation and quantitative PCR (ChIP-qPCR)

ChIP-qPCR analysis was performed as previously described [9]. In brief, NPC cells were fixed in 1% formaldehyde for 10 min, and after 5 min, glycine was added at a final concentration of 0.125 M. Cells were harvested with lysis buffers in the ChIP kit (BersinBio, China) according to the manufacturer's instructions. Chromatin was sonicated with SCIENTZ-950E (Scientz Biotechnology, China) to prepare chromatin suspensions of DNA fragments 200-600 bp in length. Immunoprecipitation reactions were performed with antibodies against Nrf2 or TAF4 (Abcam) overnight at 4 ℃.

Normal rabbit IgG was used as the negative control. After capture with protein A/G-magnetic beads, immunoprecipitated chromatin was de-cross-linked, and coimmunoprecipitated DNA fragments were purified for qPCR analysis using the following primer pairs: human SLC7A11 promoter region, forward, 5′-TTGAGCAACAAGCTCCTCCT-3′, and reverse, 5′-CAAACCAGCTCAGCTTCCTC-3′.

References

1. Li F, Zhao X, Sun R, Ou JX, Huang JY, Yang NY, et al. EGFR-rich extracellular vesicles derived from highly metastatic nasopharyngeal carcinoma cells accelerate tumour metastasis through PI3K/AKT pathway-suppressed ROS. J Extracell Vesicles. 2020; 10.
2. Dieterle ME, Sola-Riera C, Ye C, Goodfellow SM, Mittler E, Kasikci E, et al. Genetic depletion studies inform receptor usage by virulent hantaviruses in human endothelial cells. Elife. 2021; 10.
3. Goji T, Takahara K, Negishi M, Katoh H. Cystine uptake through the cystine/glutamate antiporter xCT triggers glioblastoma cell death under glucose deprivation. J Biol Chem. 2017; 292: 19721-32.
4. Li F, Li JY, Wang PH, Yang NY, Huang JY, Ou JX, et al. SARS-CoV-2 spike promotes inflammation and apoptosis through autophagy by ROS-suppressed PI3K/AKT/mTOR signaling. Bba-Mol Basis Dis. 2021; 1867.
5. Kinjyo I, Bragin D, Grattan R, Winter SS, Wilson BS. Leukemia-derived exosomes and cytokines pave the way for entry into the brain. J Leukocyte Biol. 2019; 105: 741-53.
6. Hong H, Lin X, Xu Y, Tong T, Zhang J, He H, et al. Cadmium induces ferroptosis mediated inflammation by activating Gpx4/Ager/p65 axis in pancreatic beta-cells. Sci Total Environ. 2022; 849: 157819.
7. Chanthammachat P, Dharmasaroja P. Metformin restores the mitochondrial membrane potentials in association with a reduction in TIMM23 and NDUFS3 in MPP+-induced neurotoxicity in SH-SY5Y cells. EXCLI J. 2019; 18: 812-23.
8. Wang G, Liu S, Wang L, Meng L, Cui C, Zhang H, et al. Lipocalin-2 Promotes Endoplasmic Reticulum Stress and Proliferation by Augmenting Intracellular Iron in Human Pulmonary Arterial Smooth Muscle Cells. Int J Biol Sci. 2017; 13: 135-44.
9. Ye P, Mimura J, Okada T, Sato H, Liu T, Maruyama A, et al. Nrf2- and ATF4-dependent upregulation of xCT modulates the sensitivity of T24 bladder carcinoma cells to proteasome inhibition. Mol Cell Biol. 2014; 34: 3421-34.
